# Identification and functional validation of lactylation-related hub genes in idiopathic pulmonary fibrosis based on multi-omics analysis

**DOI:** 10.1186/s12931-026-03783-3

**Published:** 2026-07-04

**Authors:** Ming Yue, Rumei Luan, Dongyan Ding, Xiao Hu, Jie Tan, Meng Zhao, Lin Zhu, Qianfei Xue, Junling Yang

**Affiliations:** 1 Department of Respiratory Medicine, The Second Hospital of Jilin University, Changchun, China; 2 Department of Respiratory Medicine, Shandong First Medical University Affiliated Provincial Hospital, Jinan, China; 3 Department of Respiratory Medicine, The 958 Hospital of Chinese PLA/Jiangbei Campus, The First Affiliated Hospital of Army Medical University, Chongqing, China; 4 Department of Respiratory Medicine, Sichuan Provincial People’s Hospital, Chengdu, China; 5 Hospital of Jilin University, Changchun, China

## Abstract

**Background:**

Idiopathic pulmonary fibrosis (IPF) is a progressive, irreversible interstitial lung disease (ILD) characterized by high mortality and poorly understood pathogenesis. Lactylation, a recently identified post-translational modification (PTM), is closely associated with inflammatory responses and tissue fibrosis in various diseases. However, the expression patterns and potential roles of lactylation, as well as the key regulatory genes involved, have not been fully elucidated in IPF. This study aimed to identify lactylation-related hub genes in IPF through bioinformatics analysis and preliminary functional validation, to explore their therapeutic potential, and to evaluate their binding affinity with clinical drugs, providing a theoretical basis for future research on lactylation mechanisms and targeted interventions in IPF.

**Methods:**

Transcriptomic and single-cell sequencing datasets from lung tissues of IPF patients and healthy controls (HC) were obtained from the Gene Expression Omnibus (GEO) database. Weighted Gene Co-expression Network Analysis (WGCNA) was performed to identify IPF-related gene modules. Lactylation-associated hub genes were determined by integrating lactylation-related gene sets with protein-protein interaction (PPI) network analysis. Seurat software and UMAP clustering were employed to identify cell-specific localization of hub genes in lung tissues. Preliminary functional validations, including immunohistochemistry (IHC), Western blotting (WB) for pan-lactylation detection, and gene knockdown assays, were conducted in vitro and in vivo. The CIBERSORT algorithm was applied to analyze immune cell infiltration profiles in IPF tissues. Molecular docking analyses were performed to assess the binding affinity of clinical antifibrotic drugs (pirfenidone and curcumin) with proteins encoded by hub genes.

**Results:**

DDX3X, BCLAF1, and NCL were identified as lactylation-related hub genes in IPF and were specifically expressed in macrophages/neutrophils, ciliated epithelial cells, and lung fibroblasts/macrophages, respectively. The level of pan-lactylation was significantly elevated in IPF lung tissues and TGF-β1-stimulated cellular models. These three hub genes were significantly upregulated in IPF tissues compared with HC, and individual knockdown of each gene significantly inhibited alveolar epithelial-mesenchymal transition (EMT) and human fetal lung fibroblast (HFL1) activation. In addition, these hub genes were closely associated with immune microenvironment remodeling in IPF. Pirfenidone and curcumin demonstrated favorable binding affinity with the proteins encoded by these genes.

**Conclusion:**

The lactylation-related hub genes DDX3X, BCLAF1, and NCL are specifically expressed in distinct cell types within IPF lung tissues, and their upregulation may promote IPF progression by mediating alveolar epithelial EMT, fibroblast activation, and profibrotic immune infiltration, highlighting their potential as therapeutic targets for IPF. Aberrant lactylation activation may contribute to IPF pathogenesis. Pirfenidone and curcumin show promising binding affinity with these hub gene-encoded proteins, providing a theoretical basis for further investigation into lactylation-targeted therapeutic strategies in IPF.

**Graphical Abstract:**

**Figure:**
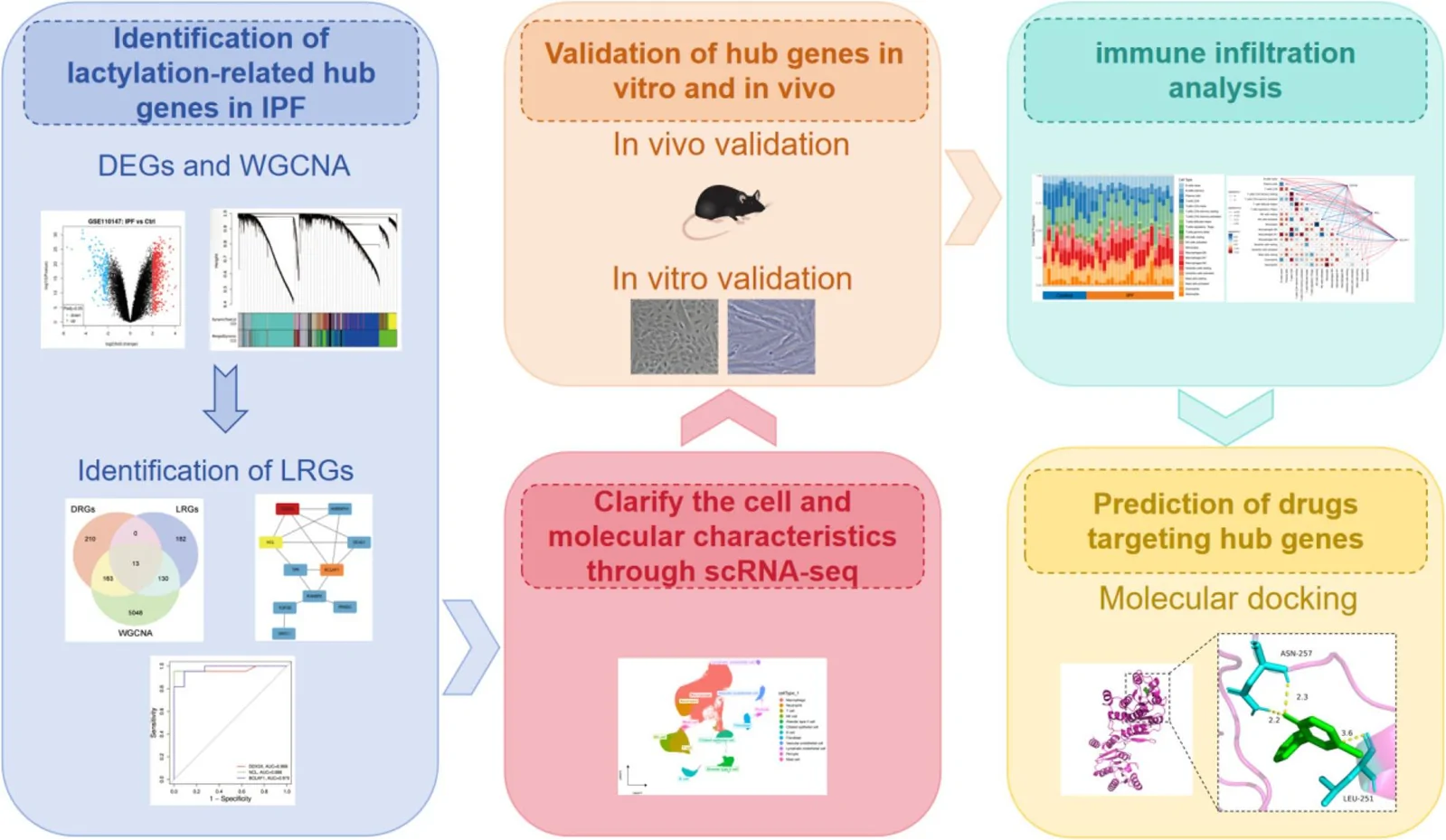

**Supplementary Information:**

The online version contains supplementary material available at https://doi.org/10.1186/s12931-026-03783-3.

## Introduction

Idiopathic pulmonary fibrosis (IPF) is a chronic, progressive interstitial lung disease (ILD) of unknown etiology, with an estimated global annual incidence of approximately 0.9 to 13 cases per 100,000 population [1]. Its core pathological feature is the usual interstitial pneumonia (UIP) pattern, which can be definitively diagnosed through lung biopsy or supported by characteristic radiological features identified via high-resolution computed tomography (HRCT). Progressive fibrotic scarring of lung tissue is a hallmark clinicopathological manifestation associated with disease advancement [2]. Among the mechanisms driving pulmonary fibrosis, epithelial-mesenchymal transition (EMT) of alveolar type II (AT II) cells is a critical process, typically occurring in areas with extracellular matrix (ECM) deposition. During EMT, epithelial cells lose their intrinsic phenotype, acquire mesenchymal characteristics, and promote fibroblast differentiation into myofibroblasts. These cellular changes establish a persistent profibrotic microenvironment, ultimately accelerating pulmonary fibrosis progression [3]. As the disease advances, patients gradually develop respiratory failure, resulting in a notably high mortality rate. Although antifibrotic medications are used clinically, their efficacy is limited to slowing disease progression rather than reversing established fibrosis, with most patients experiencing a median survival of less than 5 to 10 years post-diagnosis [4]. The limitations of current therapeutic strategies underscore the urgency to identify novel regulatory targets and intervention pathways for IPF. Recent bioinformatics and mechanistic studies have focused on identifying potential biomarkers, shared genetic signatures, hub gene networks, and key fibrogenic molecules involved in fibrotic disorders through methods such as machine learning, ferroptosis profiling, cross-disease genetic correlation analyses, multi-omics integration, and paracrine-mediated fibrotic signaling analyses [5–11].

Lactic acid, the end-product of glycolysis, functions as both a central substrate in cellular energy metabolism and a pivotal signaling molecule involved in diverse physiological and pathological processes. It is widely implicated in biological processes including energy metabolism and cellular signal transduction [12]. Lactic acid circulates through the bloodstream, accumulating within various vital organs, and regulates cellular energy metabolism and redox homeostasis through intracellular and intercellular lactate shuttle mechanisms [13]. In 2019, Zhang et al. demonstrated that lactic acid serves as a key substrate for a novel epigenetic modification, histone lysine lactylation, as verified by mass spectrometry analyses of core histones from human and mouse cells [14]. Lactylation is a ubiquitous and reversible post-translational modification (PTM), characterized by the covalent attachment of lactyl groups to lysine residues on histone tails or non-histone proteins. Initially identified in histones, lactylation has since been detected on non-histone proteins, where it modulates protein function and thereby affects multiple biological processes [15]. Importantly, an intrinsic relationship exists between lactic acid and lactylation, with lactic acid serving as an essential precursor for lactylation. Under physiological conditions, lactate homeostasis maintains basal lactylation activity. Conversely, under pathological conditions such as hypoxia and metabolic reprogramming, elevated expression of glycolytic enzymes and lactate transporters results in abnormal lactate accumulation, providing ample substrates for lactylation and significantly enhancing global lactylation levels. This substrate-driven association constitutes the central mechanism underlying metabolic-epigenetic crosstalk mediated by lactic acid and lactylation [16], laying the molecular foundation for their involvement in the pathogenesis of hypoxia-related diseases.

Gas exchange impairment caused by progressive decline in pulmonary function in IPF patients induces a characteristic hypoxic microenvironment in the lungs. This hypoxic microenvironment not only drives cellular metabolic reprogramming but also provides an essential prerequisite for aberrant activation of lactylation. Accumulating evidence indicates that lactylation exerts critical profibrotic effects during pulmonary fibrosis progression [17]. The regulatory mechanisms of lactylation are characterized by complex multi-cellular and multi-pathway interactions and are closely associated with core pathological processes of pulmonary fibrosis [18]. Studies have demonstrated that IPF-associated metabolic reprogramming induces abnormal upregulation of lactylation. By remodeling chromatin structure and regulating effector molecule activity, lactylation mediates key pathological processes, including fibroblast activation and excessive ECM deposition, thereby accelerating pulmonary fibrosis progression [19]. At the epithelial cell level, histone lactylation directly drives alveolar epithelial cell EMT by activating the TGF-β/Smad2/3 and VEGFA/ERK pathways, exacerbating fibrotic damage in lung tissue [20]. In fibroblasts, histone H3K9 lactylation upregulates METTL3 expression, which, by mediating m^6^A modification and nuclear export of lncRNAs, promotes fibroblast-to-myofibroblast transition (FMT) and further enhances the profibrotic phenotype [21]. At the immune regulatory level, lactate secreted by glycolytically active lung myofibroblasts can mediate histone lactylation at promoter regions of profibrotic genes in macrophages via p300. This promotes the transformation of macrophages into a profibrotic phenotype and facilitates intercellular communication during fibrogenesis [22], consistent with the correlation between hub genes and macrophage subsets observed in the immune infiltration analysis conducted in this study. Furthermore, in arsenic exposure-associated IPF, lactate secreted by myofibroblasts can be taken up by alveolar epithelial cells and converted to lactyl-CoA, significantly increasing lactylation modifications such as Kla and H3K18la. This process subsequently activates TGF-β1 secretion by regulating the transcription of m^6^A reader proteins, thereby promoting FMT progression and highlighting the critical role of epithelial-myofibroblast crosstalk in lactylation-mediated IPF pathogenesis [23]. Collectively, existing studies indicate that lactylation targets multiple cell types, including alveolar epithelial cells, fibroblasts, and macrophages, contributing to pulmonary fibrosis by regulating core pathological processes such as EMT, FMT, and immune microenvironment remodeling. However, the downstream regulatory genes and precise molecular networks of lactylation remain incompletely elucidated. Therefore, identifying key molecular targets of lactylation involved in regulating phenotypic transitions in pulmonary fibrosis-associated cells may provide essential theoretical support for developing novel precision therapy strategies.

Although previous multi-omics studies on IPF have primarily focused on conventional differential gene screening and phenotypic characterization at the phenomenological level [24], few studies have systematically investigated IPF from the perspective of lactylation modifications combined with single-cell profiling and comprehensive functional validation in vivo and in vitro. To address this gap, the present study constructed a multi-dimensional analytical framework to further elucidate the potential molecular and cellular characteristics of IPF.

In recent years, the rapid advancement of multi-omics technologies has driven a paradigm shift in research into the mechanisms underlying pulmonary fibrosis [25]. The integrated application of transcriptome analyses and single-cell sequencing has facilitated systematic exploration of disease-specific molecular events in pulmonary fibrogenesis [26]. At the transcriptional level, weighted gene co-expression network analysis (WGCNA) efficiently identified lactylation-related hub gene sets and key molecular clusters that regulate pulmonary fibrosis progression, providing a preliminary basis for subsequent functional validation and mechanistic investigation. At the cellular level, we first elucidated the cell-specific expression patterns of each gene in the identified hub gene set by integrating publicly available single-cell sequencing datasets with UMAP analysis. Additionally, we assessed pan-lactylation levels in mouse lung tissues using immunohistochemistry (IHC) and verified aberrantly increased pan-lactylation in mouse lung tissues, A549 cells, and HFL1 cells via Western blotting (WB). These results provided direct experimental evidence of abnormal lactylation activation during pulmonary fibrosis from both in vivo and in vitro perspectives and supported subsequent functional validation and mechanistic exploration of hub genes. We then conducted functional experiments on these hub genes to validate their regulatory roles in core pathological processes of pulmonary fibrosis, including EMT, FMT, and ECM deposition, preliminarily clarifying their potential contributions to lactylation-mediated fibrogenesis. Subsequently, immune cell infiltration analysis was performed to further investigate the intrinsic relationship between hub genes and the immune microenvironment, suggesting their possible immunomodulatory mechanisms. In addition, molecular docking was used to predict the binding interactions of the identified hub genes with pirfenidone (a clinical therapeutic agent for IPF) and curcumin (a natural bioactive compound). This approach highlighted the potential of these hub genes as druggable targets from a structural biology perspective and provided theoretical support for future research into relevant therapeutic strategies.

Collectively, this study established a comprehensive research framework: Lactylation-related hub genes were screened via transcriptomic WGCNA; cell-specific expression patterns were analyzed through single-cell UMAP analysis; functional experiments were conducted both in vitro and in vivo, including pan-lactylation detection via IHC in mouse lung tissues and WB in mouse lung tissues, A549 cells, and HFL1 cells, confirming aberrant lactylation activation during pulmonary fibrosis; immune cell infiltration was further analyzed; and molecular docking validation was performed to examine interactions between hub genes and pirfenidone or curcumin. Specifically, this study focused on three hub genes (DDX3X, BCLAF1, and NCL) and systematically explored their functional roles in lactylation-mediated IPF progression, offering novel insights into key targets and potential therapeutic sites for lactylation-mediated pulmonary fibrosis and providing a theoretical foundation for subsequent studies.

## Materials and methods

### Gene expression datasets

The gene expression dataset GSE110147 was obtained from the Gene Expression Omnibus (GEO) database (https://www.ncbi.nlm.nih.gov/geo/). This dataset comprised 22 IPF lung tissue samples and 11 normal lung tissue samples, analyzed using the Affymetrix Human Gene 1.0 ST Array (Affymetrix, Inc., Santa Clara, CA, USA; Platform GPL6244). In addition, a total of 325 lactate-related genes (LRGs) were identified and retrieved from the Molecular Signatures Database (MSigDB, http://www.gsea-msigdb.org/gsea/index.jsp) for subsequent correlation analyses [27].

### Screening and preprocessing of Differentially Expressed Genes (DEGs)

The online GEO2R analytical tool from the GEO database was used to perform comparative analyses of gene expression profiles between the IPF group and the normal control group to identify DEGs. This analysis utilized the limma R package following these procedures: a linear model was constructed, and the empirical Bayes method was applied to shrink the variance of gene expression. The moderated t-test was used to evaluate gene expression differences between groups. The Benjamini & Hochberg (BH) method was employed for correction of raw P-values to control the false discovery rate (FDR). Gene annotation information was obtained from standardized annotation data provided by the National Center for Biotechnology Information (NCBI). The selection criteria for DEGs were set as |log_2_FC| ≥ 2 and adjusted P-value < 0.05.

### WGCNA

WGCNA was performed using the WGCNA package in R. The initial dataset included 20,329 genes and 33 samples. To ensure the reliability of results, genes were filtered for expression stability; those with a low expression standard deviation (≤ 0.01) were removed. After low-expression gene filtering, no eligible genes were excluded, and all 33 samples and 20,329 genes were retained for subsequent WGCNA analysis. Subsequently, the soft threshold selection was conducted, and the optimal soft threshold was set at 16 based on the scale-free network fitting principle. Using this threshold, an adjacency matrix was constructed and converted into a Topological Overlap Matrix (TOM). Dynamic tree cutting was used to cluster genes into co-expression modules with similar expression patterns. Modules with eigengene correlations greater than 0.25 were merged. Genes within each module showing correlation coefficients above 0.3 with module eigengenes and the strongest association with the IPF phenotype were identified as IPF-related signature genes. An intersection analysis of signature genes, DEGs, and LRGs was performed using an online bioinformatics analysis platform (https://bioinformatics.com.cn/), ultimately identifying lactylation-related differentially expressed genes (LRDGs) associated with IPF.

### GO functional and KEGG pathway enrichment analyses

To characterize the biological functions and potential regulatory pathways of LRDGs, Gene Ontology (GO) functional enrichment and Kyoto Encyclopedia of Genes and Genomes (KEGG) pathway enrichment analyses were conducted on LRDGs using the Metascape online tool (https://metascape.org/gp/index.html) [28] and the online bioinformatics analysis platform (https://bioinformatics.com.cn/) [29–31].

### PPI network analysis and hub gene screening of LRDGs

The STRING online database (https://string-db.org/) [32] was utilized to construct a protein-protein interaction (PPI) network for LRDGs, with a threshold interaction score set greater than 0.40. The results generated by STRING were visualized using Cytoscape software (Version 3.9.1). The CytoHubba plugin was employed to rank network nodes based on the Maximum Clique Centrality (MCC) algorithm, and the top three genes with the highest MCC scores were selected as hub genes.

### Single-cell RNA sequencing data analysis

The single-cell RNA sequencing dataset (GSE136831) was obtained from the GEO database. This dataset included 28 normal control samples and 32 IPF disease samples. Data normalization was performed using the Seurat package in R, following these detailed procedures: cells were assessed using the total number of unique molecular identifiers (UMIs) per cell, the number of expressed genes, and the proportion of mitochondrial genes as quality control metrics. Abnormal cells deviating more than three median absolute deviations (MADs) from the median were excluded using the MAD method. Additionally, DoubletFinder software (Version 2.0.4) was applied to eliminate doublet contamination. Subsequently, a sequential series of processes was conducted, including LogNormalize normalization, cell cycle scoring using CellCycleScoring, variable gene selection via FindVariableFeatures, and batch effect correction using ScaleData. Principal component analysis (PCA) was performed through the RunPCA function for dimensionality reduction. Finally, cell type annotation was completed using SingleR software based on the CellMarker and PanglaoDB databases, supplemented by relevant published literature.

### Bleomycin (BLM)-induced pulmonary fibrosis mouse model and experimental design

All animal experiments in this study were approved by the Institutional Animal Care and Use Committee of the Basic Medical College, Jilin University (Approval No.: 2025651) and conducted in strict accordance with relevant guidelines for laboratory animal care and use. Specific pathogen-free (SPF) male C57BL/6 mice (aged 8 weeks, weighing 20–21 g) were purchased from Changchun Yisi Laboratory Animal Technology Co., Ltd. (Changchun, China), with the animal production license number SCXK (Ji) 2023–0002. Before experimentation, all mice were acclimatized for one week in an SPF-grade animal facility under controlled environmental conditions: temperature 22 ± 2 °C, relative humidity 50 ± 10%, and a 12-hour light/dark cycle. Mice had free access to standard chow and sterile water. To establish the pulmonary fibrosis model, mice were anesthetized via inhalation of isoflurane (CAS No.: 26675–46 − 7, Sigma-Aldrich) and intratracheally instilled with sterile normal saline containing BLM (5 mg/kg body weight). The control group received an equal volume of sterile normal saline. Immediately after instillation, mice were vertically positioned and gently rotated for 1 min to ensure uniform distribution of the instillate within the lung parenchyma. On day 22 post-instillation, all mice were euthanized for subsequent sample collection and analysis.

### Morphological and histological analyses

After euthanasia, the left lung lobes were immediately harvested and fixed in 4% paraformaldehyde solution for 48 h. Fixed lung tissues underwent gradient dehydration, paraffin embedding, and were serially sectioned (4 μm thickness). Hematoxylin-eosin (HE) staining and Masson’s trichrome staining were performed to evaluate histopathological alterations, and the stained sections were examined using an Olympus light microscope (Olympus, Japan) at 400× magnification. The Ashcroft scoring system [33] and the Szapiel scoring system [34] were respectively applied to quantitatively assess pulmonary fibrosis severity and inflammation in lung tissues.

### Hydroxyproline (HYP) detection

Hydroxyproline (HYP) serves as a reliable indicator of collagen metabolism and pulmonary fibrosis severity. HYP content was measured using a hydroxyproline detection kit (Cat. No.: #A030–2 − 1, Nanjing Jiancheng Bioengineering Institute, China), following detailed experimental procedures previously described by our research group [35].

### Immunohistochemical staining (IHC)

The primary antibodies used in this study included: anti-DDX3X (Huamei Biotechnology Co., Ltd., diluted 1:200), anti-BCLAF1 (Boyan Biotechnology Co., Ltd., diluted 1:100), anti-NCL (Wanlei Biotechnology Co., Ltd., diluted 1:200), and anti-pan-lactylation (Huanan Biotechnology Co., Ltd., diluted 1:200) antibodies. All experimental procedures were performed according to methods previously published by our research group [35].

### Cell culture and establishment of fibrotic cell model

The human type II alveolar epithelial cell line A549 (Cat. No.: #TCH-C116, Haixing Biotechnology Co., Ltd., China) was cultured in RPMI 1640 medium (Cat. No.: #abs9484, Absin Biotechnology Co., Ltd., Shanghai) supplemented with 10% fetal bovine serum (FBS, Cat. No.: #abs972, Absin Biotechnology Co., Ltd., Shanghai) and 1% penicillin-streptomycin mixture. The human embryonic lung fibroblast cell line HFL1 (Cat. No.: #TCH-C200, Haixing Biotechnology Co., Ltd., China) was cultured in Ham’s F-12 K medium (Cat. No.: #abs9563, Absin Biotechnology Co., Ltd., Shanghai) supplemented with the same serum and antibiotic mixture. Both cell lines were routinely maintained in a humidified incubator at 37 °C with 5% CO2. At 70%-80% confluency, the medium was replaced with serum-free medium for 12 h of synchronization. To establish a fibrotic cell model, cells were stimulated with transforming growth factor-β (TGF-β) at a final concentration of 10 ng/mL for 24 h, according to previously reported methods [35, 36]. Cells without TGF-β stimulation served as controls.

### Small Interfering RNA (siRNA)-mediated gene knockdown assay

siRNA-mediated gene knockdown assays were performed separately in HFL1 and A549 cells. Double-stranded siRNAs targeting specific genes (Wuhan Jintuosi Biotechnology Co., Ltd.) were transfected into cells using KEL-R transfection reagent (Cat. No.: #KC127-1.5, Junding Biotechnology Co., Ltd., Shanghai). After transfection, cells were treated with TGF-β1 (10 ng/mL) for 24 h to establish a fibrotic cell model. The siRNA sequences used were as follows: siDDX3X: sense 5′-GAUUCGUAGAAUAGUCGAATT-3′, antisense 5′-UUCGACUAUUCUACGAAUCTT-3′; siNCL: sense 5′-CACAAGGAAAGAAGACGAATT-3′, antisense 5′-UUCGUCUUCUUUCCUUGUGTT-3′; siBCLAF1: sense 5′-GGAAAUGGAUCUAGUCGAUTT-3′, antisense 5′-AUCGACUAGAUCCAUUUCCTT-3′.

### WB

WB was performed as previously described by our research group [35]. HFL1 and A549 cells were collected, rinsed with pre-chilled PBS, and lysed on ice for 30 min in RIPA lysis buffer containing protease/phosphatase inhibitors (Cat. No.: #P0013B, Beyotime Biotechnology, Shanghai, China). Mouse lung tissues were fully homogenized with the same batch of the above lysis buffer, followed by centrifugation at 12,000×g for 15 min at 4 °C to collect the supernatant. Protein concentration was determined using a BCA Protein Assay Kit (Cat. No.: #ZJ102, Yeasen Biotechnology, Shanghai, China). 30 µg of total protein was loaded and separated by 8%–12% SDS-PAGE gel electrophoresis, then transferred onto a PVDF membrane (Cat. No.: #IPVH00010, Merck Millipore, Germany). The membrane was blocked with 5% non-fat milk-TBST for 1 h at room temperature, and subsequently incubated with primary antibodies at 4 °C overnight. After thorough washing with TBST, the membrane was incubated with secondary antibodies (Table S2) for 1 h at room temperature. Chemiluminescence signals were detected using an Enhanced ECL Kit (Cat. No.: #BL520B, Biosharp, China), and the gray values of protein bands were quantitatively analyzed using ImageJ software (Bio-Rad, USA).

### Cell scratch assay

A549 and HFL1 cells were separately seeded into 6-well culture plates at a density of 1 × 10^6^ cells per well and cultured until reaching approximately 90% confluency. Uniform scratches were created horizontally on the cell monolayer using a 20 µL pipette tip, and cells were gently rinsed twice with serum-free medium to remove detached cells and debris. Subsequently, siRNA targeting specific genes were transfected into cells. Following transfection, TGF-β1 was added, and cells were incubated for 24 h. Images were captured at 0 h (initial time point) and 24 h after induction using an inverted microscope. Scratch width was measured using ImageJ software, and scratch healing rates were calculated to assess cell migration ability.

### Immune cell infiltration analysis

CIBERSORT, a deconvolution algorithm based on linear support vector regression, quantitatively estimates the relative proportions of 22 human immune cell subtypes from gene expression matrices [37]. Immune cell infiltration analysis was performed with a significance level set at *P* < 0.05 to evaluate differences in immune cell composition between IPF patients and healthy individuals. Additionally, correlations between lactylation-related hub genes and infiltrating immune cells were analyzed to explore potential regulatory relationships.

### Molecular docking

Molecular docking simulations were conducted to predict binding affinity and conformational interactions between key active components and core target proteins. The crystal structures of pirfenidone and curcumin, selected as key active ligands, were downloaded from the PubChem database. Protein structures of core targets were retrieved from the RCSB Protein Data Bank (PDB, https://www.rcsb.org). Crystal water molecules and heteroatoms were removed from the protein structures using PyMOL software, and the resulting proteins were prepared as protein receptors for molecular docking. Docking simulations were automatically performed using the online tool CB-DOCK2 (https://cadd.labshare.cn/cb-dock2), which integrates the AutoDock Vina 1.2.0 algorithm for structure-based docking.

Prior to docking, the topological similarity of protein-ligand complexes was calculated based on the BioLip database (Version 2021.09.15). Complexes with a sequence identity > 40% at template ligand-binding sites and a pocket root mean square deviation (RMSD) ≤ 4 Å were retained for docking analysis. The FitDock algorithm [38] was employed in CB-DOCK2. A binding energy < 0 kcal/mol indicates spontaneous receptor-ligand binding, with smaller values indicating stronger binding affinity. In this study, a binding energy threshold of ≤ − 5 kcal/mol was applied to identify stable receptor-ligand complexes.

### Statistical analysis

Statistical analyses were conducted using SPSS software (Version 20.0; SPSS Inc., Chicago, IL, USA) and GraphPad Prism software (Version 5.0; GraphPad Software Inc., San Diego, CA, USA). Categorical variables were presented as numbers (percentages) [n(%)], and comparisons between groups were performed using the chi-square test or Fisher’s exact test as appropriate. Normality testing was conducted first for continuous variables: variables with a normal distribution were expressed as mean ± standard deviation (x̄± SD) and were compared using the independent samples t-test, whereas non-normally distributed variables were expressed as median (interquartile range, IQR) and analyzed using the Mann-Whitney U test. Bivariate correlations were analyzed using Spearman’s correlation coefficient. The predictive value of variables was assessed using receiver operating characteristic (ROC) curve analysis, with the area under the curve (AUC) calculated. Correlation analyses between hub genes and immune cells, as well as immune checkpoint genes, utilized Pearson’s correlation coefficient or Spearman’s rank correlation test, depending on data distribution. Positive cell ratios in IHC sections were quantified using ImageJ software. A two-tailed P-value < 0.05 was considered statistically significant, with significance levels indicated as follows: *p < 0.05, **p < 0.01, ***p < 0.001, ****p < 0.0001.

## Results

### Identification of DEGs in lung tissues based on the GSE110147 dataset

**Fig. 1:**
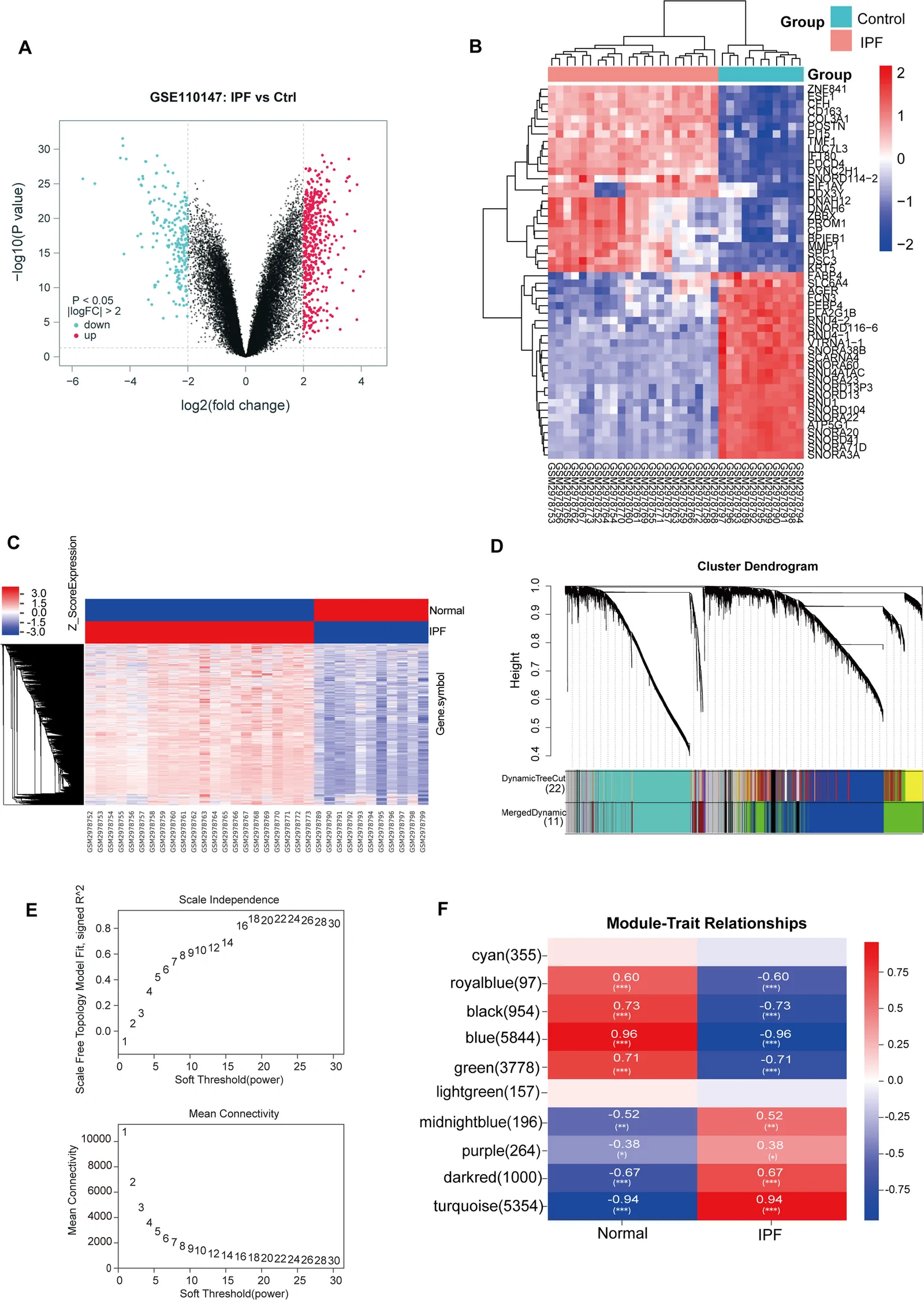
Screening of DEGs, heatmap visualization and WGCNA based on the GEO dataset GSE110147. **A** Volcano plot depicting DEGs between IPF and HC lung tissues; **B** Heatmap showing the top 25 significantly up- and downregulated DEGs; **C** Global gene expression heatmap of normal and IPF lung tissues; **D** Gene clustering dendrogram with module assignment; **E** Soft-threshold selection for WGCNA (scale independence and mean connectivity); **F** Module–trait association heatmap

To characterize gene expression profiles in lung tissues from IPF patients, DEGs between IPF patients and healthy controls (HC) were identified using the GEO2R online tool based on the GSE110147 dataset; the results were illustrated in Fig. 1A. Using selection criteria of |log_2_FC| ≥ 2 and adjusted *P* < 0.05, a total of 386 DEGs were identified, comprising 242 upregulated genes and 144 downregulated genes. The top 25 most significantly up- and downregulated DEGs underwent cluster analysis, with their expression patterns visualized as a heatmap (Fig. 1B).

### Screening of IPF-related lactylation-associated gene modules via WGCNA

To identify lactylation-associated genes closely related to IPF progression, WGCNA was performed on gene expression profiles from IPF patients and HC using the GSE110147 dataset. The optimal soft-threshold power (β = 16) was determined through scale-free network topology fitting analysis, and the gene co-expression network constructed with this parameter conformed to scale-free distribution characteristics. Gene co-expression modules were established based on the topological overlap matrix, and module-phenotype correlations were assessed. Results indicated that the turquoise module exhibited a strong correlation with the IPF phenotype (correlation coefficient = 0.94, *P* < 0.001). Correlation analyses and module-phenotype characteristics were shown in Fig. 1C-F. The turquoise module contained 5354 genes, forming the candidate gene set for subsequent lactylation-related hub gene screening. 

### Screening of LRDGs and their biological functional enrichment analysis

**Fig. 2:**
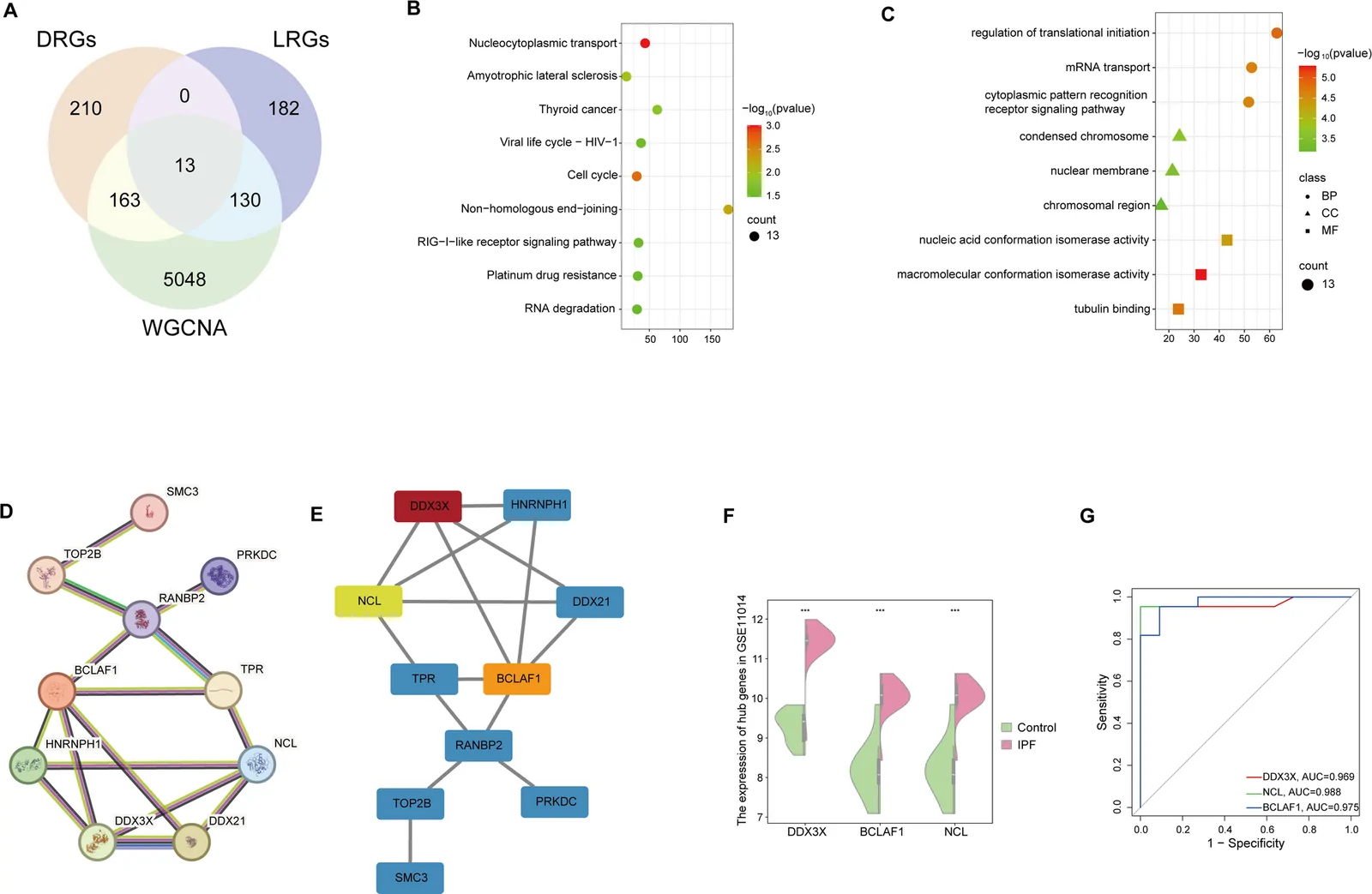
Screening and functional enrichment of LRDGs, and identification and expression profiles of hub LRDGs in IPF. **A** Venn diagram depicting intersection among DEGs, LRGs, and key WGCNA module genes; **B** KEGG pathway enrichment bubble plot of LRDGs; **C** GO functional enrichment bubble plot of LRDGs; **D** PPI network of the 13 identified LRDGs; **E** Co-expression network of hub LRDGs identified by CytoHubba; **F** Violin plots illustrating mRNA expression of DDX3X, BCLAF1, and NCL in IPF versus control lung tissues (GSE110147); **G** ROC curves evaluating the diagnostic efficiency of DDX3X, BCLAF1, and NCL in IPF

A three-set intersection analysis strategy was employed to identify LRDGs involved in IPF pathogenesis. Intersection analysis was conducted among DEGs derived from differential analysis of the GSE110147 dataset, reported LRGs, and genes from the turquoise module highly correlated with the IPF phenotype via WGCNA. This analysis identified 13 LRDGs: TOP2B, NCL, DDX3X, DDX21, HNRNPH1, LRPPRC, TPR, BCLAF1, SMC3, MPHOSPH6, BZW1, PRKDC, and RANBP2. The intersection screening process was illustrated in Fig. 2A. To further elucidate the biological functions and potential regulatory pathways of these LRDGs, KEGG pathway enrichment and GO functional enrichment analyses were performed using the online platform Metascape. GO analysis encompassed three categories: molecular function (MF), biological process (BP), and cellular component (CC), with visualization of these enrichment results presented in Fig. 2B and C. KEGG pathway enrichment analysis revealed significant enrichment of LRDGs in nucleocytoplasmic transport, amyotrophic lateral sclerosis, and thyroid cancer pathways. GO-MF analysis indicated that LRDGs were primarily involved in nucleic acid conformation isomerase activity, macromolecular conformation isomerase activity, and tubulin binding. GO-BP analysis demonstrated that these LRDGs were associated mainly with translation initiation regulation, mRNA transport, and the cytoplasmic pattern recognition receptor signaling pathway. GO-CC analysis confirmed predominant cellular localization of LRDGs in condensed chromosomes, nuclear membranes, and chromosomal regions.

### PPI network analysis

To clarify PPIs among the 13 LRDGs, a PPI network was constructed using the STRING online database (interaction score threshold > 0.4); results are shown in Fig. 2D. Subsequently, the CytoHubba plugin within Cytoscape software was used to identify hub genes. Based on the maximum clique centrality (MCC) scoring, the top three hub LRDGs (DDX3X, BCLAF1, and NCL) were identified, with detailed results illustrated in Fig. 2E.

### Expression levels of DDX3X, BCLAF1, and NCL in lung tissues from HC and IPF patients in the dataset

In lung tissue samples from the GSE110147 dataset, mRNA expression levels of the three hub LRDGs (DDX3X, BCLAF1, and NCL) were significantly upregulated in IPF patients compared to HC (Fig. 2F). ROC curve analyses showed that these genes effectively distinguished IPF from HC samples, with respective AUC values of 0.969, 0.998, and 0.975, demonstrating strong diagnostic potential for IPF (Fig. 2G).

### Analysis of cellular heterogeneity and expression characteristics of lactylation-related hub LRDGs in IPF lung tissues based on scRNA-seq

**Fig. 3:**
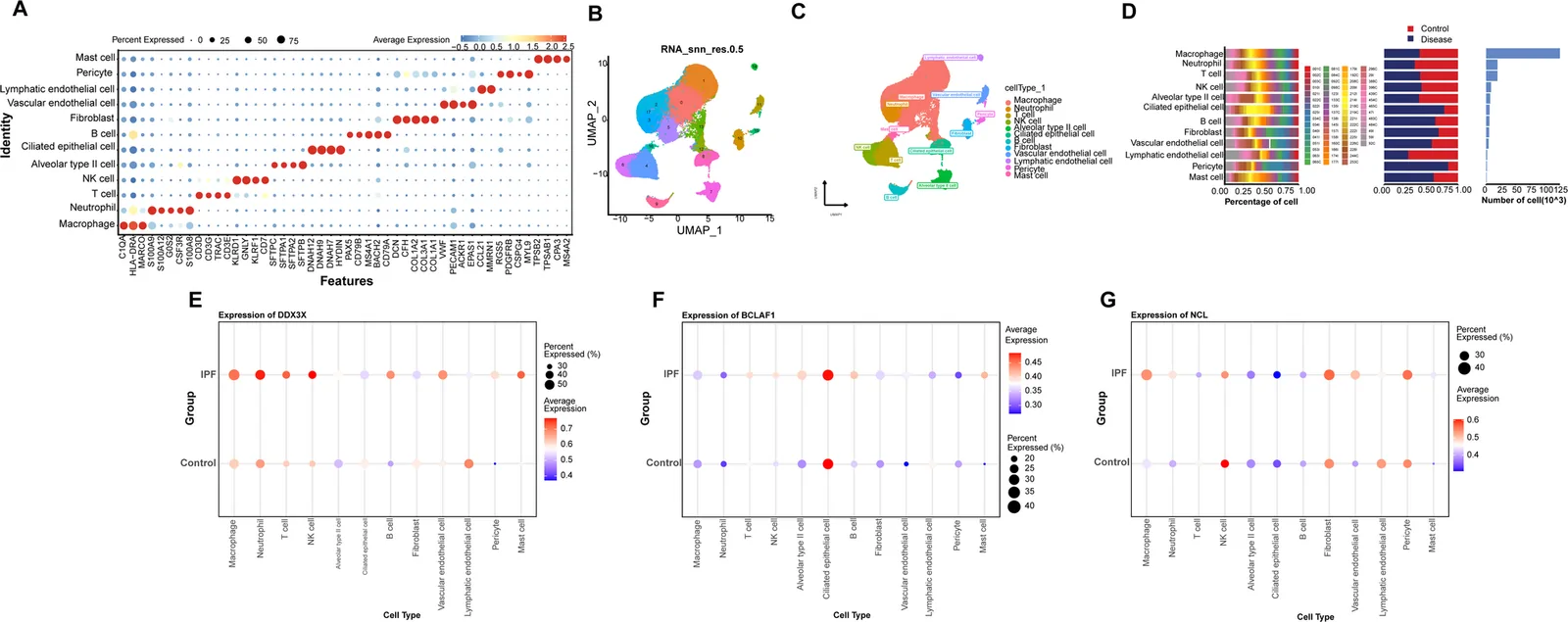
scRNA-seq analysis of lung tissues from IPF patients and HC (GSE136831 dataset). **A** Validation of marker gene expression; **B** Unsupervised clustering results; **C** Cell type annotation; **D** Analysis of cellular heterogeneity between IPF and control groups; **E**-**G** Differential expression of DDX3X, BCLAF1, and NCL across distinct cell types

To clarify the cellular localization and expression characteristics of the three hub LRDGs (DDX3X, BCLAF1, and NCL) in IPF lung tissues and to analyze cellular heterogeneity between IPF and HC lung tissues, the scRNA-seq dataset (GSE136831) obtained from the GEO database was systematically analyzed. First, the reliability of cell type-specific marker gene expression was validated using a marker gene expression dot plot, providing the basis for subsequent cell clustering and accurate annotation (Fig. 3A). Based on UMAP dimensionality reduction and clustering analysis, a total of 18 cell subclusters were identified (Fig. 3B). According to annotation criteria from the CellMarker database, PanglaoDB database, and published literature, these subclusters were further classified into 12 major cell types, including macrophages, neutrophils, T cells, natural killer (NK) cells, type II alveolar epithelial cells, ciliated epithelial cells, B cells, fibroblasts, vascular endothelial cells, lymphatic endothelial cells, pericytes, and mast cells (Fig. 3C). Subsequently, differences in cell proportions between IPF and HC lung tissues were compared. The results demonstrated significant heterogeneity in cellular composition between the two groups (all *P* < 0.05). Compared with controls, the IPF group exhibited significantly increased proportions of profibrotic immune cells (macrophages and neutrophils) and fibroblasts, which are key effector cells in fibrosis, whereas the proportions of other structural cell types, including endothelial cells, were also significantly altered (Fig. 3D). Based on these cell annotation results, expression differences of the three hub LRDGs across distinct cell types were further investigated between the IPF and control groups. Expression profiling revealed distinct cell-specific expression patterns for all three genes: DDX3X was significantly upregulated in macrophages and neutrophils in the IPF group (*P* < 0.001); BCLAF1 expression was significantly increased in ciliated epithelial cells (*P* < 0.01); and NCL was significantly upregulated in fibroblasts and macrophages (*P* < 0.001) (Fig. 3E). These findings clarified the target cell populations of hub LRDGs and provided an important basis for subsequent investigation of the regulatory network involving lactylation, hub genes, and target cells.

### Expression verification of hub genes in a BLM-induced pulmonary fibrosis mouse model

**Fig. 4:**
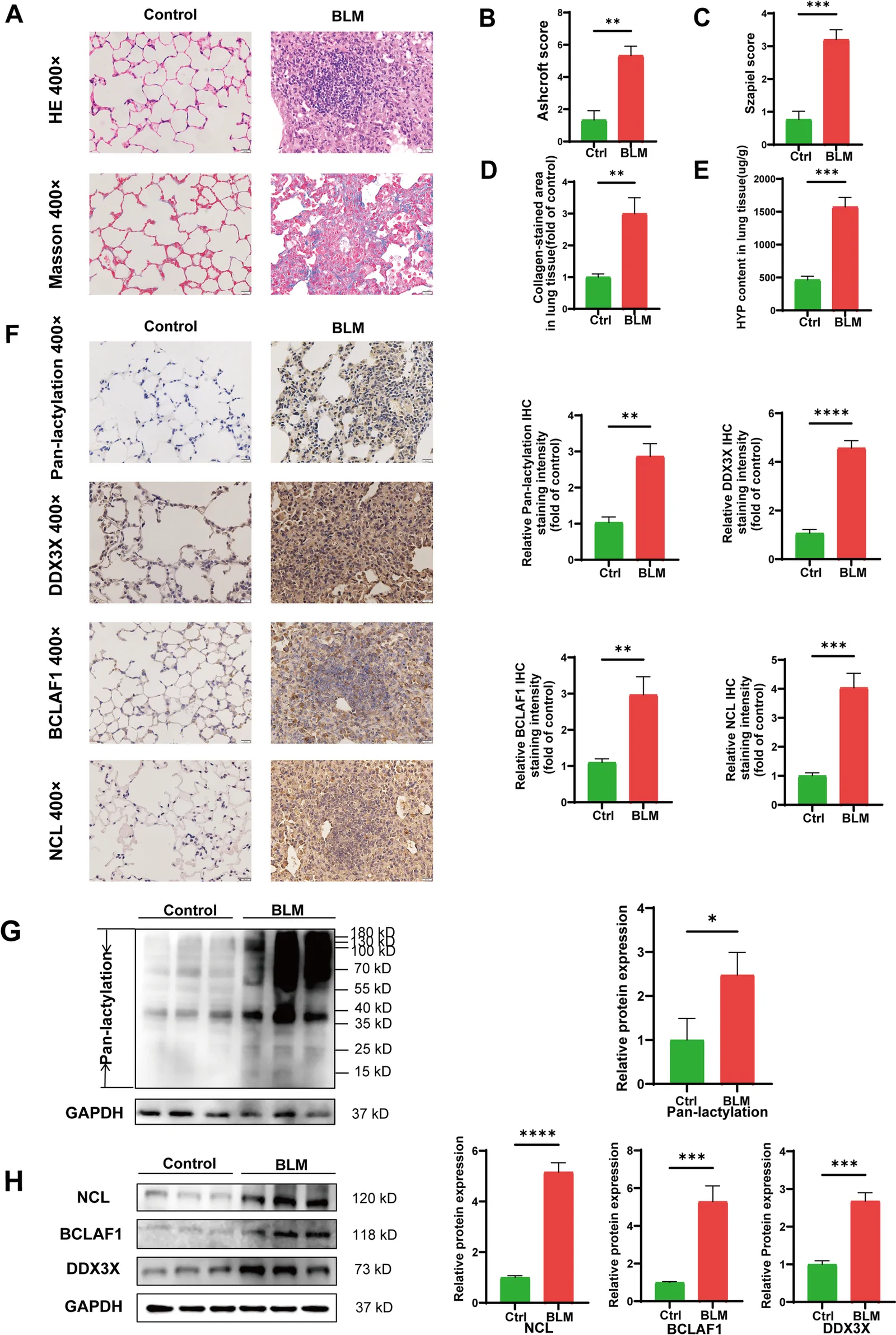
In vivo validation of hub genes in a BLM-induced pulmonary fibrosis mouse model. **A** HE and Masson staining of lung tissues from control and BLM-treated mice (×400); **B** Ashcroft fibrosis score; **C** Szapiel inflammation score; **D** Collagen content in lung tissues; **E** Hydroxyproline content in lung tissues; **F** IHC staining (×400) showing expression and localization of pan-lactylation, DDX3X, BCLAF1, and NCL; **G** WB analysis of pan-lactylation expression; **H** WB analysis of NCL, BCLAF1, and DDX3X expression

To validate the in vivo expression dynamics and cellular localization characteristics of the hub genes DDX3X, BCLAF1, and NCL during pulmonary fibrosis progression, a BLM-induced mouse model of pulmonary fibrosis was established, followed by validation experiments using histological staining, IHC, and WB. HE and Masson staining demonstrated that lung tissues from the BLM-treated group exhibited marked pathological alterations, including disrupted alveolar architecture, extensive inflammatory cell infiltration within the pulmonary interstitium, and substantial collagen deposition. In contrast, lung tissues from the control group displayed intact alveolar structures without significant inflammatory infiltration or abnormal collagen accumulation (Fig. 4A). Further quantitative analyses showed that the Ashcroft fibrosis score, Szapiel inflammation score, total collagen content, and hydroxyproline content in the BLM-treated group were all significantly higher than those in the control group (*P* < 0.05; Fig. 4B-E), confirming successful establishment of the BLM-induced pulmonary fibrosis model for subsequent gene expression validation experiments. Using this established model, pan-lactylation expression in mouse lung tissues was first evaluated by IHC to verify its abnormal activation during pulmonary fibrosis. Compared with the control group, pan-lactylation expression in the BLM-treated group was significantly elevated (*P* < 0.05), with positive signals primarily localized to pulmonary interstitial regions containing fibrotic lesions (Fig. 4F). WB analysis further confirmed that pan-lactylation levels in lung tissues from the BLM-treated group were significantly higher than those in the control group (*P* < 0.05), consistent with the IHC findings and further supporting abnormal upregulation of pan-lactylation in the BLM-induced pulmonary fibrosis model (Fig. 4G). Subsequently, IHC staining was performed to determine the cellular localization and expression patterns of proteins encoded by the three hub genes. DDX3X staining was predominantly localized in the cytoplasm [39], BCLAF1 staining was mainly concentrated in the nucleus [40], whereas NCL exhibited both nuclear and cytoplasmic localization, indicating dual localization characteristics [41]. Compared with the control group, staining intensities of all three proteins were significantly increased in lung tissues from BLM-treated mice (all *P* < 0.05), with positive signals mainly enriched in pulmonary interstitial fibrotic lesions and surrounding cells (Fig. 4F), suggesting that upregulated hub gene expression was closely associated with pulmonary fibrotic lesion formation. To further validate the IHC findings, WB analysis was performed to assess protein expression levels of DDX3X, BCLAF1, and NCL in mouse lung tissues. The results demonstrated significantly increased protein expression of all three hub genes in the BLM-treated group compared with the control group (all *P* < 0.05), consistent with the IHC results and further confirming abnormal upregulation of these hub genes in the BLM-induced pulmonary fibrosis mouse model (Fig. 4H).

### Functional validation of hub genes in TGF-β-induced fibrosis of alveolar epithelial cells

**Fig. 5:**
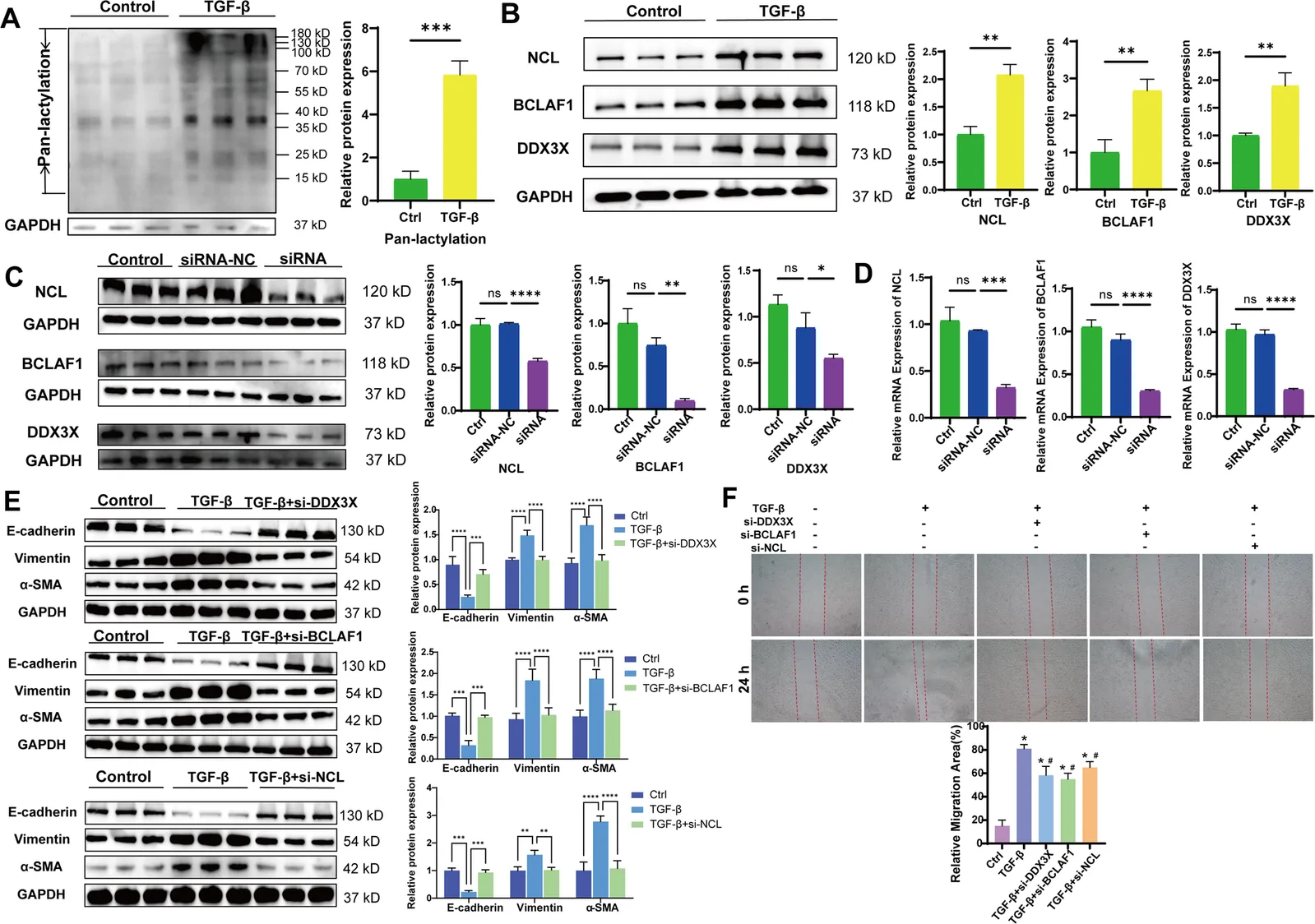
Functional validation of hub genes in TGF-β-induced fibrosis of A549 cells. **A** Pan-lactylation levels detected by WB in A549 cells stimulated with 10 ng/mL TGF-β for 24 h; **B** Protein expression of NCL, DDX3X, and BCLAF1 by WB; **C** Silencing efficiency of NCL, DDX3X, and BCLAF1 siRNAs confirmed by WB; **D** mRNA expression of hub genes after siRNA knockdown determined by qRT-PCR; **E** Expression of EMT markers (E-cadherin, Vimentin, α-SMA) after hub gene silencing assessed by WB; **F** Cell scratch assay evaluating migration ability of A549 cells at 0 h and 24 h

To further elucidate the regulatory roles of the hub genes (DDX3X, BCLAF1, and NCL) during alveolar epithelial cell fibrosis, a TGF-β-induced EMT model was established using the human alveolar type II epithelial cell line A549. By employing siRNA-mediated gene silencing, we systematically explored the effects of these hub genes on the fibrotic phenotype of A549 cells. First, we evaluated pan-lactylation levels and expression dynamics of the three hub genes following TGF-β stimulation. WB analysis revealed that treatment with 10 ng/mL TGF-β for 24 h significantly increased pan-lactylation levels (Fig. 5A) and protein expression of DDX3X, BCLAF1, and NCL (Fig. 5B), suggesting a close association between these genes and TGF-β-induced fibrotic progression in alveolar epithelial cells. To investigate their biological functions, we individually silenced DDX3X, BCLAF1, and NCL using gene-specific siRNAs in A549 cells. WB confirmed effective reduction of target protein expression (Fig. 5C), and qRT-PCR further verified significant downregulation of mRNA levels (Fig. 5D), establishing a reliable gene-silencing system for subsequent functional assays. Using this validated system, we examined how hub gene silencing affected EMT/fibrosis marker expression and cell migration. WB analysis showed that TGF-β treatment alone significantly reduced expression of the epithelial marker E-cadherin and enhanced mesenchymal markers Vimentin and α-SMA, confirming successful EMT induction in A549 cells (Fig. 5E). However, silencing of DDX3X, BCLAF1, or NCL markedly reversed this EMT phenotype by restoring E-cadherin levels and suppressing the TGF-β-induced upregulation of Vimentin and α-SMA (Fig. 5E). Cell scratch assays demonstrated that TGF-β enhanced A549 cell migration, whereas silencing each of the three hub genes significantly attenuated this effect (Fig. 5F), further supporting their pro-fibrotic roles.

### Functional validation of hub genes in TGF-β-induced fibrosis of HFL1 cells

**Fig. 6:**
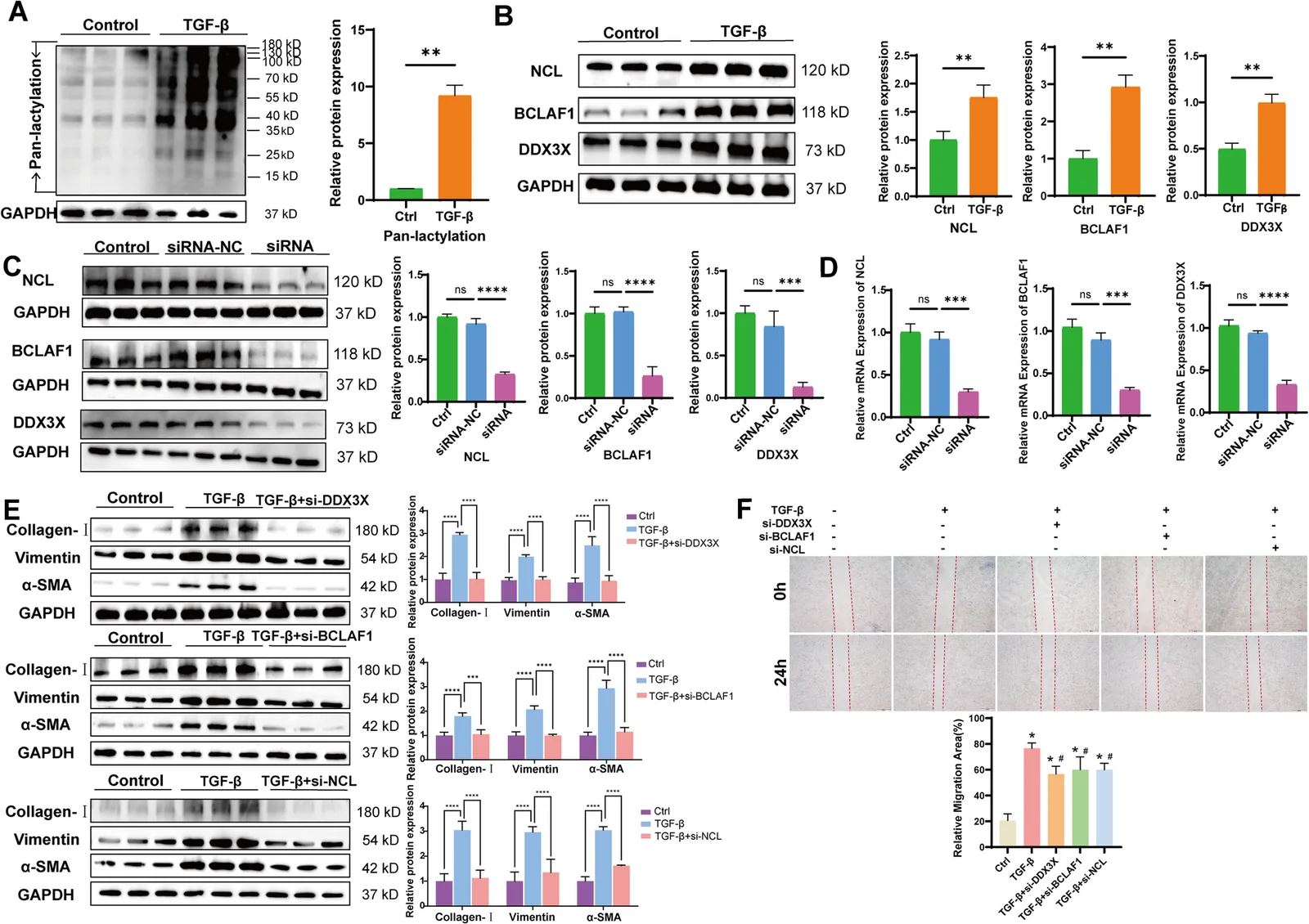
Functional validation of hub genes in TGF-β-induced fibrosis of HFL1 cells. **A** Pan-lactylation levels detected by WB in HFL1 cells stimulated with 10 ng/mL TGF-β for 24 h; **B** Protein expression of NCL, DDX3X, and BCLAF1 by WB; **C** Validation of siRNA-mediated knockdown efficiency for NCL, DDX3X, and BCLAF1 by WB; **D** mRNA expression levels of hub genes following siRNA transfection determined by qRT-PCR; **E** Expression of fibrosis markers (Collagen-I, Vimentin, α-SMA) after hub gene silencing assessed by WB; **F** Cell scratch assay evaluating migration ability of HFL1 cells at 0 h and 24 h

To further elucidate the regulatory roles of the hub genes DDX3X, BCLAF1, and NCL in fibrotic activation of lung fibroblasts, the human lung fibroblast cell line HFL1 was utilized to establish a fibroblast activation model induced by 10 ng/mL TGF-β. By combining this model with siRNA-mediated gene silencing, we systematically explored the regulatory effects and underlying mechanisms of these hub genes in HFL1 cells. Initially, pan-lactylation levels and hub gene expression were assessed following TGF-β treatment. WB analysis demonstrated significantly elevated pan-lactylation modifications (Fig. 6A) and increased protein expression of DDX3X, BCLAF1, and NCL (Fig. 6B) in HFL1 cells after 24 h of TGF-β stimulation. These findings suggest a positive correlation between abnormal pan-lactylation activation, hub gene upregulation, and TGF-β-induced fibroblast activation. To verify the functional roles of these genes, specific siRNAs targeting DDX3X, BCLAF1, and NCL were individually transfected into HFL1 cells. WB confirmed effective protein knockdown (Fig. 6C), and qRT-PCR analysis further verified significantly reduced mRNA expression levels (Fig. 6D), establishing an efficient and specific gene silencing system for subsequent experiments. Using this validated model, we investigated the regulatory impact of hub gene silencing on fibrosis-related markers. WB analysis indicated that TGF-β treatment significantly upregulated expression of fibrosis markers including Collagen-I, Vimentin, and α-SMA. Conversely, silencing each hub gene significantly reversed these TGF-β-induced effects (Fig. 6E). Additionally, cell scratch assays revealed that TGF-β significantly enhanced HFL1 cell migration, whereas silencing of each hub gene markedly inhibited this pro-migratory effect (Fig. 6F), indicating a positive regulatory role for these hub genes in fibroblast migration associated with fibrosis.

### Immune cell infiltration characteristics and correlation with hub genes

**Fig. 7:**
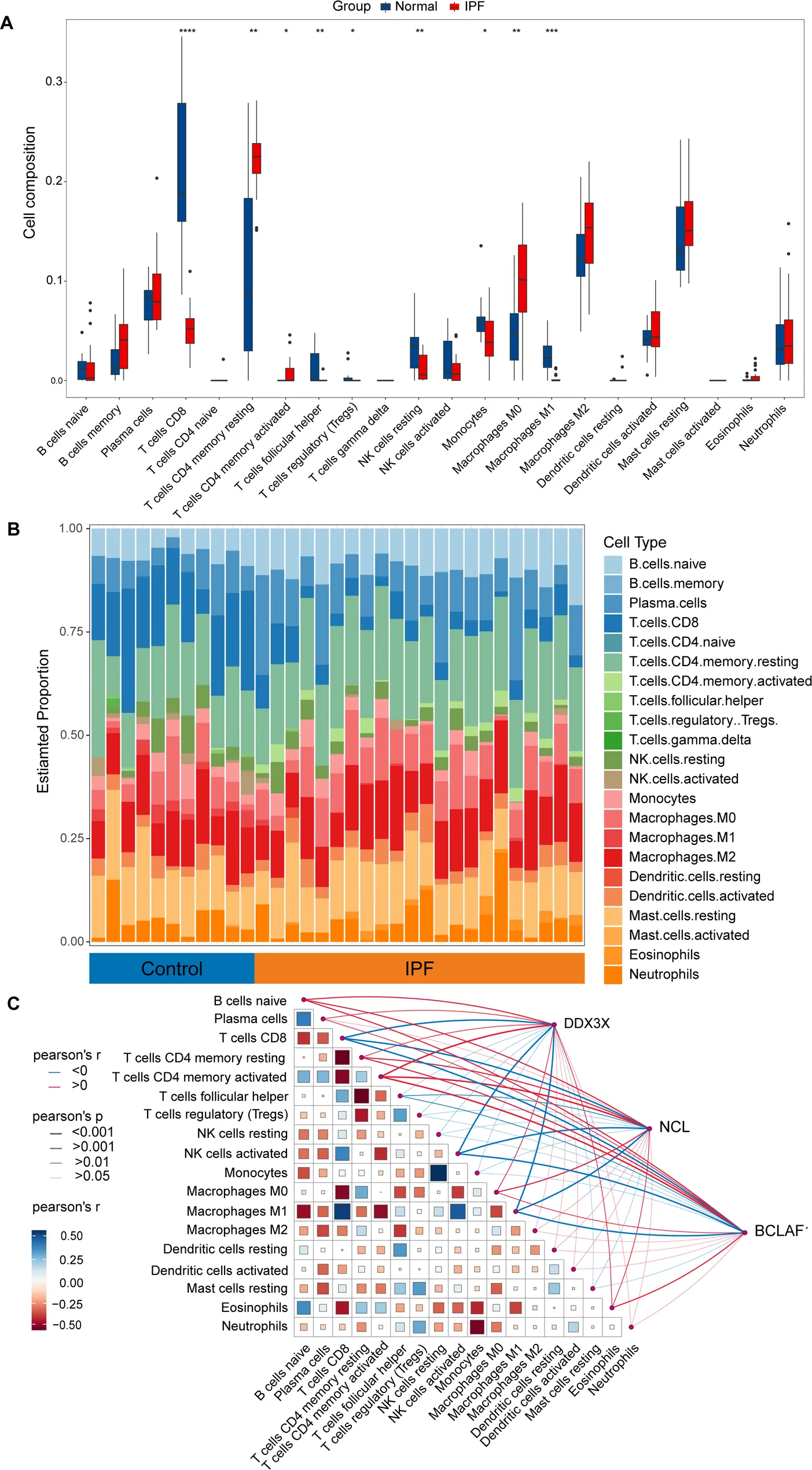
Immune cell infiltration characteristics and correlation with hub genes in lung tissues of IPF patients and HC. **A** Box plots showing infiltration differences of 22 immune cell types between the two groups; **B** Heatmap illustrating clustering characteristics of immune infiltration patterns between groups; **C** Correlation network plot revealing positive and negative correlations between immune cells and hub genes (BCLAF1, NCL, and DDX3X)

To clarify the regulatory roles of hub genes in the IPF immune microenvironment, we conducted immune infiltration analysis of the GSE110147 dataset using the CIBERSORT algorithm. Results indicated that infiltration levels of naive CD8^+^ T cells, follicular helper T cells, regulatory T cells (Tregs), resting natural killer cells, monocytes, and M1 macrophages were significantly greater in the lung tissues of HC compared with the IPF group. In contrast, the IPF group exhibited significantly higher infiltration levels of resting CD4 memory T cells, activated CD4 memory T cells, and M0 macrophages compared to controls (Fig. 7A). To intuitively illustrate overall differences in immune infiltration patterns between the groups, we generated a heatmap of immune cell infiltration. The heatmap demonstrated that IPF samples clustered distinctly into an independent branch characterized by high infiltration, clearly separating them from the low-infiltration pattern observed in the control group (Fig. 7B). This finding indicates global differences in immune cell infiltration patterns between the groups. To further elucidate the regulatory relationships between hub genes and immune cells, we constructed a butterfly plot showing correlations between immune cell infiltration and hub genes (Fig. 7C). In this plot, red lines represent positive correlations and blue lines represent negative correlations; the thickness and color intensity of lines correspond to correlation strength and statistical significance, respectively. Subsequent quantitative analysis confirmed that the three hub genes (DDX3X, BCLAF1, and NCL) exhibited a highly consistent synergistic regulatory pattern. All three genes were significantly positively correlated with activated CD4 memory T cells (*r* = 0.603–0.678, *P* < 0.001), eosinophils (*r* = 0.452–0.625, *P* < 0.001), and naive B cells (*r* = 0.402–0.509, *P* < 0.01). They were also significantly negatively correlated with CD8^+^ T cells (*r* = -0.690 to -0.802, *P* < 0.001), M1 macrophages (*r* = -0.576 to -0.712, *P* < 0.001), and activated NK cells (*r* = -0.449 to -0.556, *P* < 0.01). In addition, NCL and BCLAF1 showed significant negative correlations with follicular helper T cells (*r* = -0.364, *P* < 0.05). Each hub gene also exhibited distinct immune-regulatory characteristics: DDX3X was significantly positively correlated with resting CD4 memory T cells (*r* = 0.353, *P* < 0.05) and plasma cells (*r* = 0.348, *P* < 0.05); BCLAF1 had the strongest positive correlation with resting CD4 memory T cells (*r* = 0.530, *P* < 0.001), suggesting it may play a dominant regulatory role; NCL was significantly positively correlated not only with resting CD4 memory T cells (*r* = 0.482, *P* < 0.01) but also with M0 macrophages (*r* = 0.426, *P* < 0.05). In summary, these findings indicate that hub genes may contribute to remodeling the IPF immune microenvironment by synergistically regulating immune cell infiltration patterns, providing an essential basis for understanding the crosstalk mechanisms between immunity and fibrosis.

### Molecular docking results of hub gene-encoded proteins with anti-fibrotic drugs

**Fig. 8:**
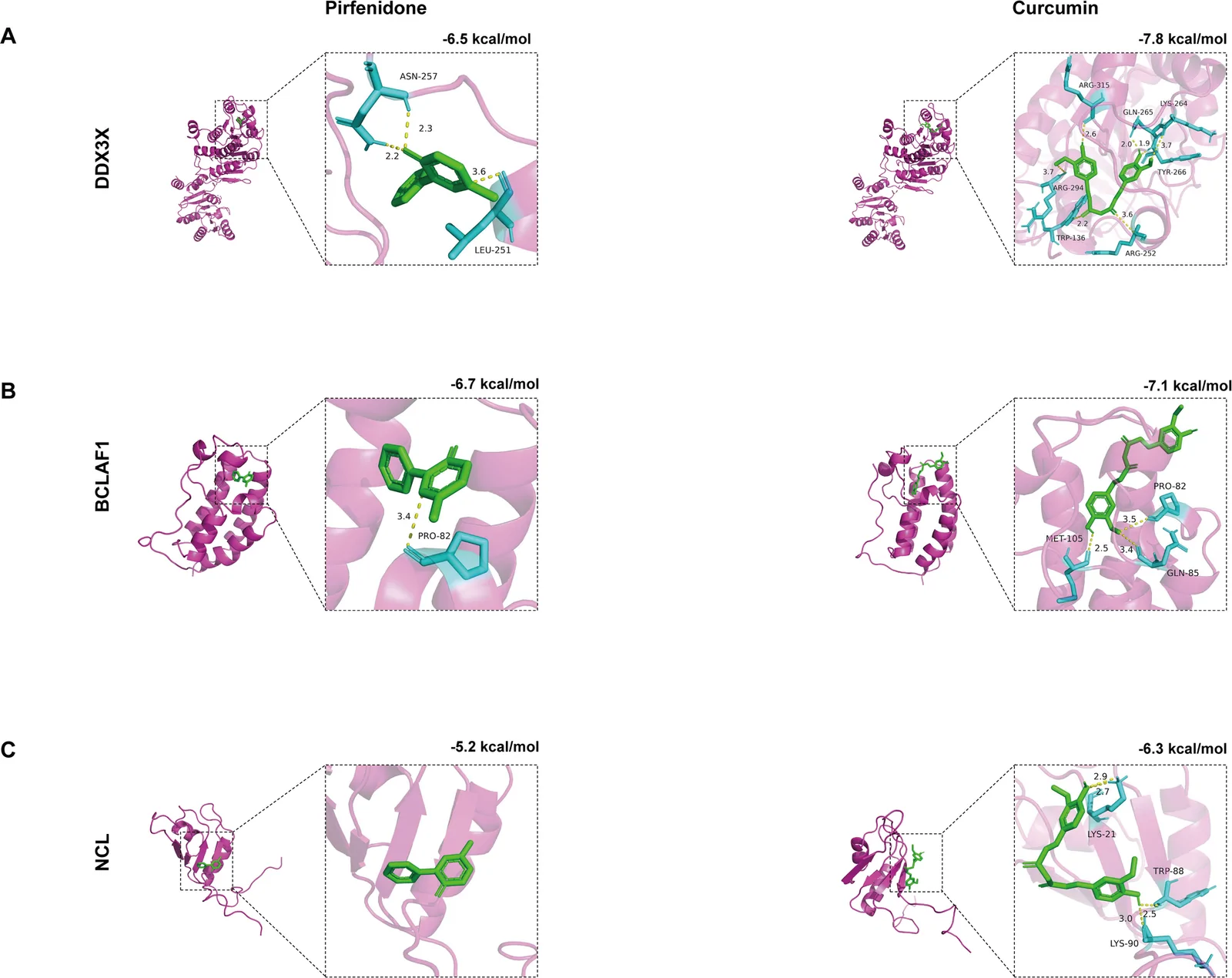
Molecular docking analysis of hub proteins (DDX3X, BCLAF1, and NCL) with pirfenidone and curcumin. **A**-**C** Docking conformations of DDX3X (PDB: 8SSW), BCLAF1 (PDB: 7RJR), and NCL (PDB: 2FC8) with pirfenidone (left) and curcumin (right). Green: drug molecules; cyan: key binding amino acid residues; yellow dashed lines: hydrogen bond interactions. Docking binding energies (kcal/mol) are indicated in the upper right

To explore the feasibility of hub gene-encoded proteins as potential targets for anti-fibrotic drugs, we selected pirfenidone (a clinically used anti-fibrotic drug) and curcumin (an anti-fibrotic active component derived from traditional Chinese medicine) for molecular docking analysis. We retrieved the crystal structures of DDX3X (PDB ID: 8SSW), BCLAF1 (PDB ID: 7RJR), and NCL (PDB ID: 2FC8) from the Protein Data Bank (PDB). Molecular docking was performed to systematically analyze binding modes and interaction characteristics between the two drugs and the aforementioned proteins. Specifically, the docking binding energy between DDX3X and pirfenidone was − 6.5 kcal/mol. The drug molecule formed stable hydrogen bonds with amino acid residues ASN-257 and LEU-251 (Fig. 8A, left). The docking binding energy with curcumin was − 7.8 kcal/mol. Curcumin bound efficiently to the protein via hydrogen bond interactions mediated by residues GLN-265, ARG-294, ARG-315, ARG-252, TYR-266, TRP-136 and LYS-264 (Fig. 8A, right). For BCLAF1, the docking binding energy with pirfenidone was − 6.7 kcal/mol. The drug molecule primarily targeted residue PRO-82, forming hydrogen bonds (Fig. 8B, left). The docking binding energy with curcumin reached − 7.1 kcal/mol; curcumin simultaneously bound to residues PRO-82, MET-105, and GLN-85, mediated by multiple hydrogen bond interactions (Fig. 8B, right). In addition, docking binding energies of NCL with pirfenidone and curcumin were − 5.2 kcal/mol and − 6.3 kcal/mol, respectively (Fig. 8C, left). Curcumin formed hydrogen bonds with residues LYS-21, TRP-88, and LYS-90, further enhancing binding stability (Fig. 8C, right). Collectively, both drugs demonstrated relatively stable interactions with the three hub proteins (all binding energies < -5 kcal/mol), suggesting that proteins encoded by hub genes could serve as promising candidate targets in anti-fibrotic drug research.

## Discussion

IPF is a rapidly progressive ILD with an extremely poor prognosis, and its clinical management has long been constrained by limitations of current therapies [42]. First-line antifibrotic agents can only slow disease progression but cannot reverse established pathological fibrotic changes [43]. Lung transplantation, on the other hand, remains limited in clinical application due to donor shortages, postoperative rejection, and other challenges. Therefore, identifying novel therapeutic targets and elucidating regulatory mechanisms are critically important to overcome current bottlenecks in IPF diagnosis and treatment [44]. In recent years, lactylation, an emerging protein PTM, has gained increasing attention for its regulatory role in fibrotic diseases [45]. This study investigated the association between lactylation and IPF, identifying and validating three hub regulatory genes (DDX3X, BCLAF1, and NCL) through multi-level experiments. These findings provide a valuable reference and potential experimental targets for further exploring the molecular networks associated with IPF pathological progression and developing targeted intervention strategies.

By integrating bioinformatics analysis and single-cell sequencing, this study first identified LRDGs via differential gene screening, followed by KEGG pathway enrichment analysis. The results indicated that these LRDGs were mainly enriched in pathways such as nucleocytoplasmic transport, amyotrophic lateral sclerosis, and thyroid cancer. Notably, the nucleocytoplasmic transport pathway is closely associated with intracellular trafficking and functional activation of key signaling molecules (e.g., TGF-β/Smad, HIF-1α) during fibrosis [46, 47]. Importantly, lactylation can modulate the binding affinity and functional activity of nucleocytoplasmic transport-related proteins (e.g., nuclear pore complex proteins and nuclear transport receptors) by regulating their lysine lactylation levels [48, 49]. This further controls the nucleocytoplasmic shuttling of pro-fibrotic signaling molecules and the transcription of downstream target genes, a mechanism highly consistent with the observed role of LRDGs in lactylation-mediated fibrosis regulation. Building upon these results, this study clarified the cell-specific expression patterns of DDX3X, BCLAF1, and NCL in IPF lung tissues, offering a novel clue for further exploring the regulatory association between lactylation and the inflammation as well as tissue remodeling in IPF.

Single-cell sequencing revealed significant disturbances in cellular composition within IPF lung tissues, characterized by increased infiltration of immune cells (e.g., macrophages and neutrophils) and abnormal proportions of structural cells (e.g., fibroblasts and endothelial cells). This finding aligns closely with typical pathological features of inflammatory activation and tissue remodeling observed in IPF [50, 51]. Differential expression profiles of the three hub genes further underscore the complexity of lactylation regulation in IPF. Specifically, DDX3X is overexpressed predominantly in infiltrating immune cells, suggesting its involvement in lactylation-mediated pro-fibrotic phenotypic transformation of immune cells and the subsequent formation of an inflammatory microenvironment initiating fibrosis. BCLAF1 is aberrantly accumulated in ciliated epithelial cells, and given that epithelial dysfunction is a pivotal early pathological event in IPF, BCLAF1 likely acts as a key mediator of lactylation-regulated EMT. NCL is upregulated in both immune cells and fibroblasts, positioning it as a potential molecular hub connecting immune inflammation and fibrosis. NCL not only amplifies inflammatory signals by modulating immune cell function but also directly contributes to fibroblast activation and fibroblast-myofibroblast transition (FMT), thus accelerating ECM deposition. These cell-type-specific expression patterns suggest that lactylation may modulate IPF progression by affecting multiple cell types and related molecular pathways, which provides a valuable reference for further exploring the regulatory network among lactylation, hub genes and target cells.

In vitro and in vivo experimental results further confirmed the pro-fibrotic functions of DDX3X, BCLAF1, and NCL, as well as their close association with lactylation. In vivo experiments, IHC and WB assays initially verified abnormal activation of lactylation in BLM-induced IPF mouse lung tissues. IHC staining demonstrated prominent enrichment of pan-lactylation-positive signals in pulmonary interstitial fibrotic lesions and inflammatory infiltration regions. WB quantification further confirmed a significant upregulation of pan-lactylation modification levels in fibrotic lung tissues. Meanwhile, expression levels of the three core proteins were significantly elevated in the IPF mouse lung tissues. Notably, regions with high core protein expression exhibited a distinct spatial correlation with pan-lactylation enrichment areas and IPF-characteristic inflammatory infiltrates and fibrotic lesions. This finding not only confirmed the consistency of the abnormal mRNA expression trends identified by single-cell sequencing at the protein level but also provided direct in vivo evidence of spatial and functional associations between pan-lactylation and the three hub genes in pulmonary fibrosis. Quantitative WB results further substantiated increased core protein expression during fibrosis, offering additional in vivo evidence supporting the involvement of hub genes in pulmonary fibrosis. In vitro, we selected A549 and HFL1 cells, two key cell types involved in IPF pathology, to establish TGF-β1-induced fibrosis models. WB analysis consistently showed significant upregulation of pan-lactylation modification levels in both cell types following TGF-β1 stimulation. This result aligned closely with the abnormal activation pattern of pan-lactylation observed in the in vivo mouse model, confirming the conserved regulatory role of lactylation in IPF fibrotic progression across different experimental systems. Although this model selection differs from the natural cellular expression patterns of hub genes identified through single-cell sequencing, it provides clear complementary research logic. On the one hand, A549 and HFL1 cells represent classic models that mediate the pathological sequence of alveolar epithelial cell injury, abnormal fibroblast activation, and ECM deposition. These models allow precise analysis of the regulatory effects of hub genes on fibrotic phenotypes under controlled in vitro conditions. In contrast, the pro-fibrotic effects of immune cells and ciliated epithelial cells depend on complex in vivo microenvironments that are difficult to fully replicate in vitro. On the other hand, the in vitro experiments verified functional conservation of hub genes, demonstrating that even genes predominantly expressed in immune or ciliated epithelial cells maintain active fibrosis-related signaling pathways in alveolar epithelial cells and fibroblasts. This allows preliminary functional deductions through classic cellular models. Experimental results indicated that core protein expression significantly increased during fibrosis. Specific knockdown of DDX3X, BCLAF1, and NCL resulted in reduced expression of fibrotic marker proteins, such as α-SMA and collagen I, and decreased migration capacities of A549 and HFL1 cells. This directly confirmed the pro-fibrotic roles of these three genes in IPF pathology. By clarifying the expression characteristics of hub genes and abnormal activation of lactylation in fibrotic tissues through in vivo experiments, and verifying their key regulatory functions via in vitro cell models, this study successfully established a comprehensive research loop, from in vivo expression profiling to in vitro functional validation.

Integrating current research progress, we further clarified potential regulatory mechanisms between the hub genes and lactylation, highlighting directions for future in-depth investigations. For DDX3X, existing studies have confirmed its pro-fibrotic role in BLM-induced pulmonary fibrosis models. It promotes disease progression by regulating RNA translation, inhibiting apoptosis, and enhancing fibroblast proliferation, adhesion, and migration. Additionally, it forms a positive feedback loop with TGF-β1 and neuraminidase 3 (NEU3), amplifying pro-fibrotic signaling [52]. Currently, studies directly associating DDX3X with lactylation remain scarce. However, the regulatory role of DDX3X in stress granule (SG) assembly in hepatocytes [53], demonstrates significant functional overlap with lactylation-mediated SG formation pathways. A study by Li et al. [54] demonstrated that lactylation modification at the K177 residue of high mobility group box 1 (HMGB1) under hypoxic stress serves as a critical upstream signal, driving nuclear export of T-cell intracellular antigen-related protein (TIAR) and initiating SG assembly. Mutation of this lysine residue to arginine (K177R) completely abolished this stress-response pathway. Meanwhile, Yang et al. [53] further clarified that DDX3X, as an RNA helicase, participates in constructing the SG core structure by regulating RNA folding and sorting, making it an indispensable component of SG assembly. Thus, existing research initially supports a regulatory framework connecting lactylation, SG assembly, and DDX3X function. However, whether DDX3X itself undergoes lactylation, including the identification of specific modification sites and underlying regulatory mechanisms, requires further experimental validation. BCLAF1 is a multifunctional protein involved in various biological processes, including apoptosis regulation, DNA damage repair, transcription regulation, and the formation of membrane-less organelles. Recent studies have identified its critical role in fibrosis through regulation of stress granule dynamics, metabolic reprogramming, and inflammatory signaling pathways, with potential links to lactylation. Under hypoxic conditions, enhanced glycolysis leads to lactic acid accumulation. Lactylation prevents degradation of hypoxia-inducible factor-1α (HIF-1α) and enhances its transcriptional activity, enabling HIF-1α binding to the BCLAF1 promoter, thus activating its transcription. Activated BCLAF1 subsequently stabilizes HIF-1α, forming a positive feedback loop. Concurrently, BCLAF1 upregulates key glycolytic enzymes, further increasing lactic acid production and establishing a hypoxia-centered metabolic-transcriptional regulatory network involving lactylation, HIF-1α, and BCLAF1 [40, 55]. The TGF-β/SMAD signaling pathway finely regulates BCLAF1 expression and function at both transcriptional and splicing levels [56]. As a central pro-fibrotic mediator in pulmonary fibrosis, TGF-β1-mediated BCLAF1 upregulation and subtype switching may facilitate fibrosis progression by promoting fibroblast activation, ECM deposition, and EMT. In systemic sclerosis-related interstitial lung disease (SSc-ILD), BCLAF1 functions as a key transcription factor involved in early disease onset and sustained fibrosis [57]. NCL, a multifunctional nucleolar protein confirmed to undergo lactylation, acts as a central molecule in fibrosis regulation. In diabetic nephropathy-related mesangial fibrosis, NCL serves as a core pro-fibrotic target, with its abnormal expression and function driving mesangial cell proliferation and fibrosis [58]. TGF-β1 induction elevates NCL expression, which subsequently promotes the expression of plasminogen activator inhibitor-1 (PAI-1) by stabilizing its mRNA, thereby advancing fibrosis progression [59]. In silica-induced pulmonary fibrosis, abnormal overexpression of NCL contributes to DNA damage, EMT, and collagen deposition, making it a pivotal target for PolyG-mediated anti-silicosis fibrosis [60]. NCL forms complexes with the long non-coding RNA CYTOR and the Sam68 protein, activating the nuclear factor-κB (NF-κB) signaling pathway and regulating EMT, thus serving as a molecular hub in malignant tumor progression [61]. Additionally, NCL binds to the mRNA of EMT markers, such as fibronectin and matrix metalloproteinases (MMPs), enhancing their stability and translational efficiency. This binding activates the fibronectin/integrin signaling axis, driving ECM remodeling and further promoting EMT [62]. As a key regulator of ribosome biosynthesis, NCL physically interacts with the RNA-binding protein LIX1L within the nucleolus, forming the LIX1L-NCL-rRNA synthesis axis to mediate rRNA synthesis. This pathway represents a novel mechanism for EMT activation; inhibiting NCL expression or blocking NCL-mediated rRNA synthesis reverses enhanced EMT-related functions caused by LIX1L overexpression [63]. Existing studies confirm lysine 477 on NCL as a specific lactylation target of the acetyltransferase p300, with modification occurring in response to excessive cellular glycolytic activation [64]. This finding provides essential theoretical support for the NCL-lactylation association identified in this study.

To further elucidate the regulatory relationships among hub genes, lactylation, and the pathological processes in IPF, this study conducted immune infiltration analysis, confirming significant immune microenvironment remodeling in IPF lung tissues, closely associated with the regulatory functions of DDX3X, BCLAF1, and NCL. In the lung tissues of the HC group, anti-inflammatory immune subsets centered around M1 macrophages, CD8^+^ T cells, and follicular helper T cells predominated, thereby maintaining immune homeostasis. In contrast, IPF tissues exhibited a characteristic immune subset imbalance, with significantly elevated infiltration of activated CD4 memory T cells, M0 macrophages, and resting CD4 memory T cells, forming a pro-inflammatory and pro-fibrotic immune microenvironment. These results align well with previously reported classic features of immune imbalance in IPF [65], further supporting immune microenvironment disorder as a pivotal pathological mechanism driving IPF progression and providing phenotypic evidence for immune regulatory mechanisms mediated by the hub genes. Notably, DDX3X, BCLAF1, and NCL showed synergistic yet distinct functional characteristics in IPF immune regulation: all three genes positively regulated infiltration of activated CD4 memory T cells, eosinophils, and naive B cells, while negatively regulating functional activity of CD8^+^ T cells, M1 macrophages, and activated NK cells. By disrupting the balance between pro- and anti-inflammatory immune responses, these three genes accelerated the inflammatory-fibrotic cascade. Regarding functional differentiation, BCLAF1 demonstrated the strongest correlation with resting CD4 memory T cells, indicating its potential involvement in activation and initiation of early immune memory responses in IPF, thus laying a foundation for inflammatory responses. NCL displayed consistent expression trends with M0 macrophages, suggesting a possible regulatory role in macrophage phenotypic polarization, potentially influenced by lactylation. This provides direction for future exploration of the lactylation-hub gene-immune cell regulatory axis.

Based on the immunoregulatory and pro-fibrotic roles of these hub genes, molecular docking experiments were conducted to analyze the binding characteristics of pirfenidone (a first-line clinical anti-fibrotic drug) and curcumin (a plant-derived candidate drug) with DDX3X, BCLAF1, and NCL. This analysis aimed to clarify the structural basis of targeted binding between these hub genes and anti-fibrotic drugs, thereby bridging hub gene functional research and drug development. The docking results demonstrated that both compounds formed stable complexes with functional domains of the three hub proteins, with curcumin exhibiting greater binding affinity than pirfenidone. Such stable binding interactions may depend on specific interactions with key amino acid residues in the target proteins, potentially related to their structural stability and functional activities. This study provides mechanistic insights from a molecular structural perspective into the potential molecular targets of pirfenidone in IPF, suggesting pirfenidone may exert therapeutic effects by targeting these hub genes and regulating the lactylation-hub gene pathway. Curcumin, with its relatively stronger binding affinity, also shows promising potential for targeting this pathway. It may influence the pro-fibrotic properties of hub proteins at the molecular level, thereby providing preliminary theoretical support for further mechanistic exploration and the development of novel anti-IPF candidate compounds.

### Limitations and future perspectives

This study has several limitations. First, only A549 and HFL1 cells were employed in vitro, without functional validation using immune cells naturally expressing high levels of the hub genes. Future studies should expand to include primary immune cell models to enhance cell-specific functional analysis. Second, only one IPF mouse model was adopted for in vivo experiments. The absence of further validation with clinical samples limits the clinical relevance of the observed association between hub genes and lactylation. Third, the precise lactylation modification sites of hub genes were not identified. Subsequent research should use mass spectrometry analysis, site-directed mutagenesis, and related techniques to explore these modification sites. Fourth, molecular docking provided only preliminary computational predictions; further validation through cellular drug intervention and molecular binding assays is required to confirm the docking results. Fifth, the current study mainly focuses on bioinformatic screening, expression profiling and phenotypic verification at tissue and cellular levels. The upstream and downstream signaling pathways as well as the detailed molecular regulatory network governing lactylation mediated by these hub genes were not fully explored.

Future studies should precisely identify lactylation modification sites on the core hub genes and systematically clarify the upstream and downstream signaling pathways and sophisticated molecular regulatory network by which these genes mediate lactylation and IPF progression. Additional clinical samples and IPF patient cohorts will improve the generalizability and translational relevance of the findings. Primary alveolar epithelial cells and corresponding immune cell models should be incorporated for functional validation aligned with the cell-specific characteristics identified via single-cell RNA sequencing. Additionally, drug intervention studies and molecular binding validation experiments should be conducted to confirm molecular docking results and further establish the therapeutic potential of candidate compounds.

## Conclusion

This study identified three lactylation-related hub genes (DDX3X, BCLAF1, and NCL) through comprehensive experimental approaches. Based on single-cell data analysis, it characterized the cell-type-specific expression patterns of these hub genes in IPF tissues and explored their potential roles in immune microenvironment regulation. WB and IHC analyses revealed increased lactylation levels in bleomycin-induced mouse pulmonary fibrosis models and TGF-β1-treated A549/HFL1 cell models. The three hub genes exhibited elevated expression in IPF-related samples, indicating that their upregulation may be associated with increased lactylation in IPF. Furthermore, preliminary functional experiments using A549 cells (alveolar epithelial cells) and HFL1 cells (fibroblasts) demonstrated that knocking down the three hub genes partially inhibited the EMT process in A549 cells and significantly suppressed fibroblast activation in HFL1 cells. These findings suggest that the identified hub genes may have pro-fibrotic roles in pulmonary fibrosis. Collectively, this study provides new insights into the potential association between lactylation and immune microenvironment remodeling in IPF, as well as the fibrotic phenotypic transformation of alveolar epithelial cells and fibroblasts (including EMT and fibroblast activation). It also supplies molecular clues for further exploration of anti-fibrotic regulatory mechanisms and targeted research, thus offering important theoretical implications. Future research will focus on identifying precise lactylation modification sites of the core hub genes, validating clinical implications of the lactylation-hub gene regulatory axis in IPF patient cohorts, elucidating the regulatory impact of hub gene knockdown on EMT and fibroblast activation, and conducting molecular binding assays to consolidate these findings. This approach is expected to provide foundational experimental evidence supporting future mechanistic investigations and translational research in IPF.

## Supplementary Information

Supplementary Material 1.

## Data Availability

The datasets generated during and/or analysed during the current study are available in the Gene Expression Omnibus database, https://www.ncbi.nlm.nih.gov/geo/, and GSE110147 and GSE136831.
