# Supplementary material for: Identification and functional validation of lactylation-related hub genes in idiopathic pulmonary fibrosis based on multi-omics analysis
Source: Respir Res. 2026 Jul 4;27:420. doi: 10.1186/s12931-026-03783-3 (PMC13715148; doi:10.1186/s12931-026-03783-3)

Figure 4G
n=3
Pan-lactylation

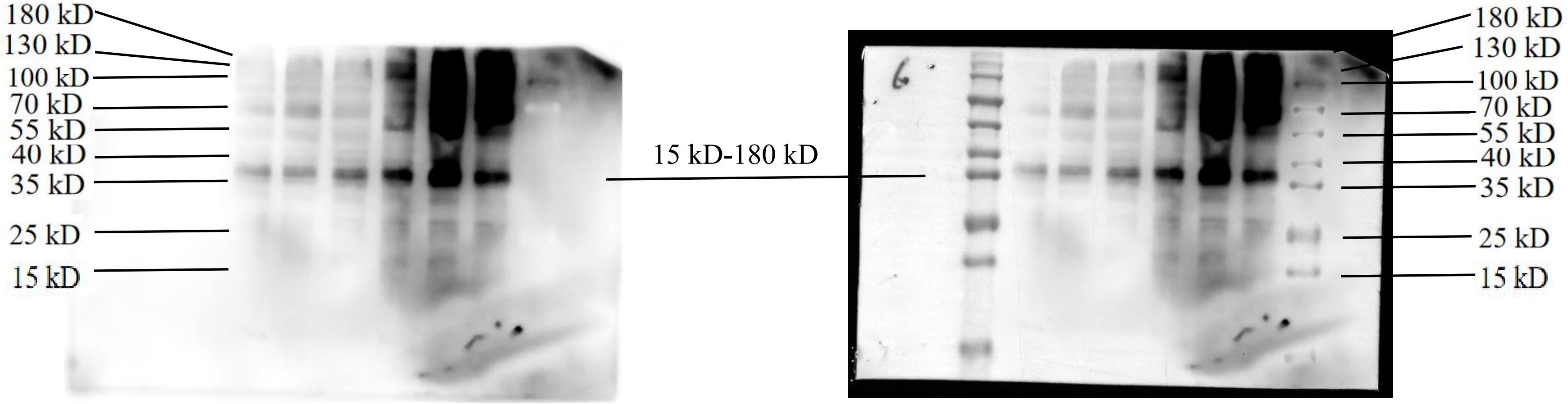

GAPDH

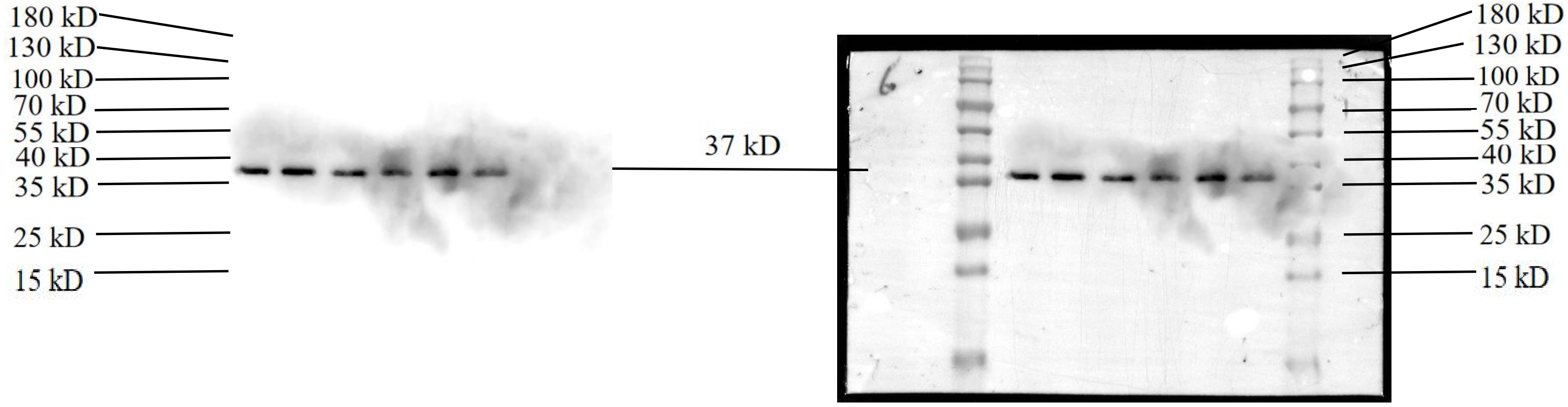

Figure 4H

n=3

NCL

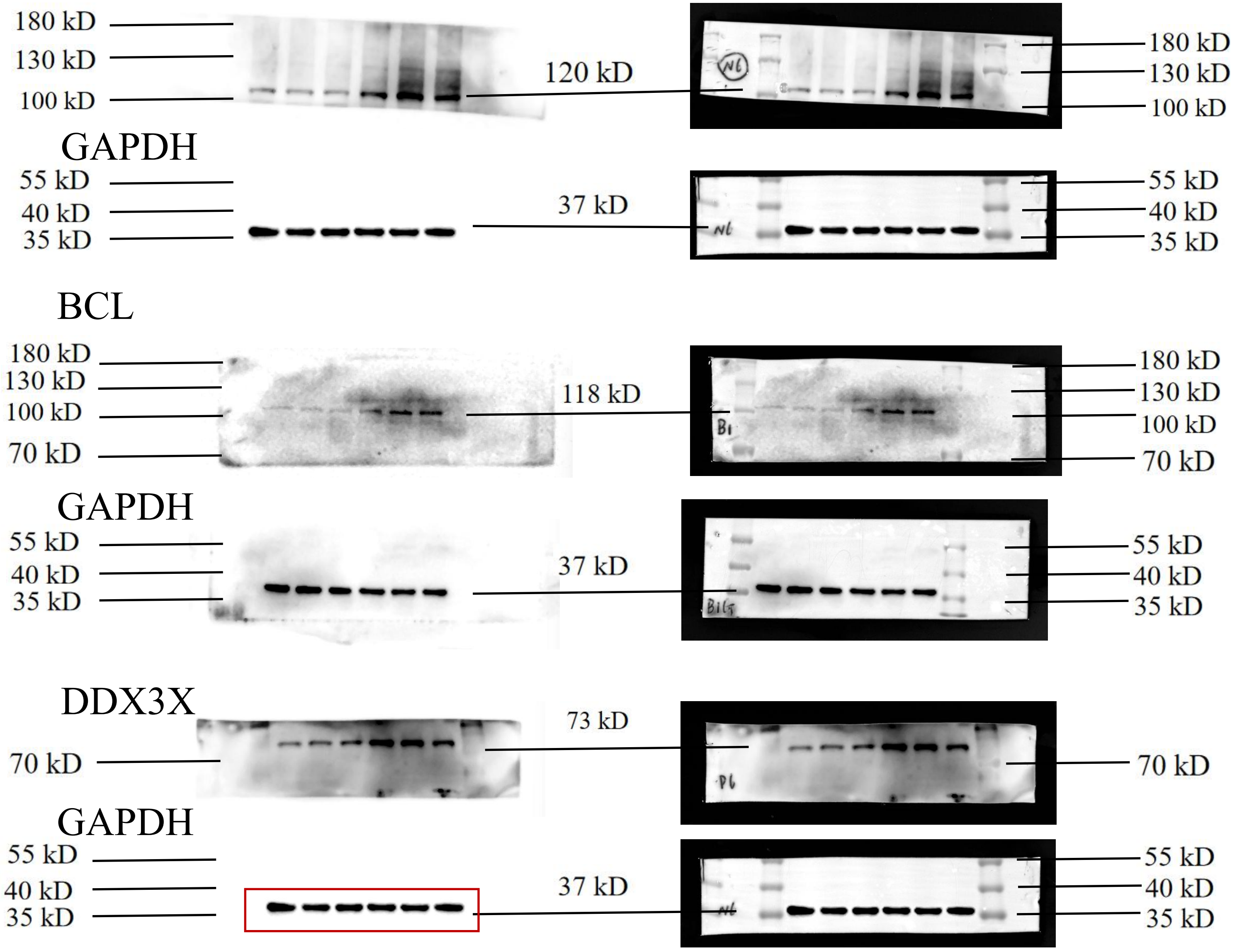

Figure 5A

n=3

Pan-lactylation

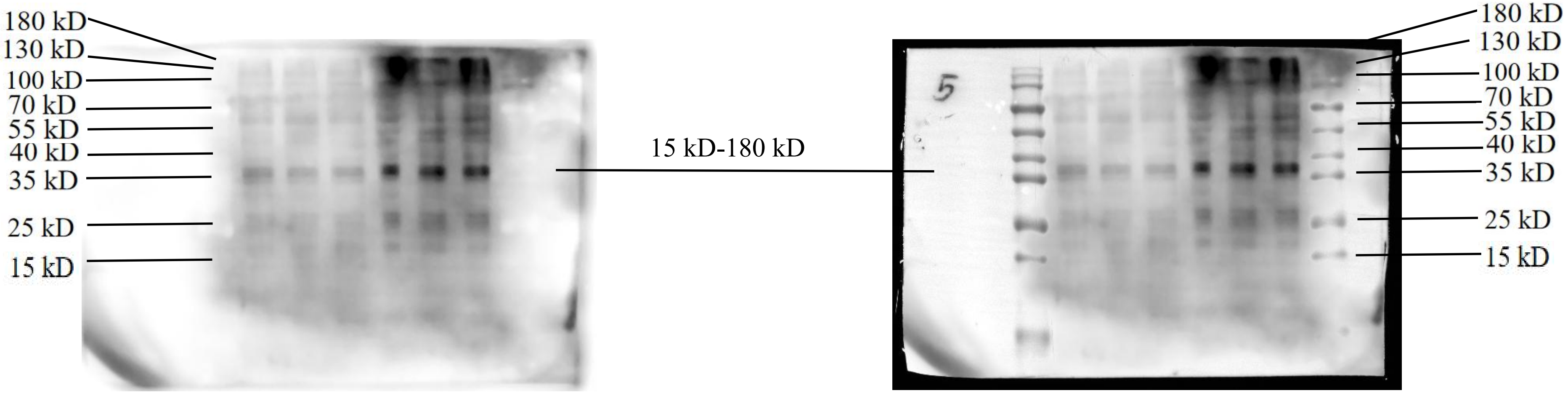

GAPDH

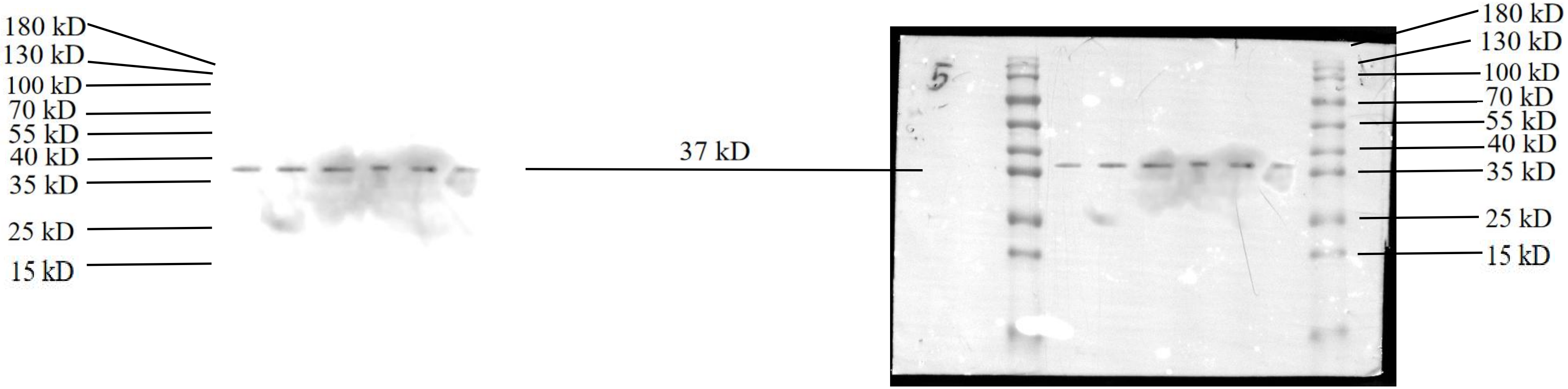

Figure 5B
n=3

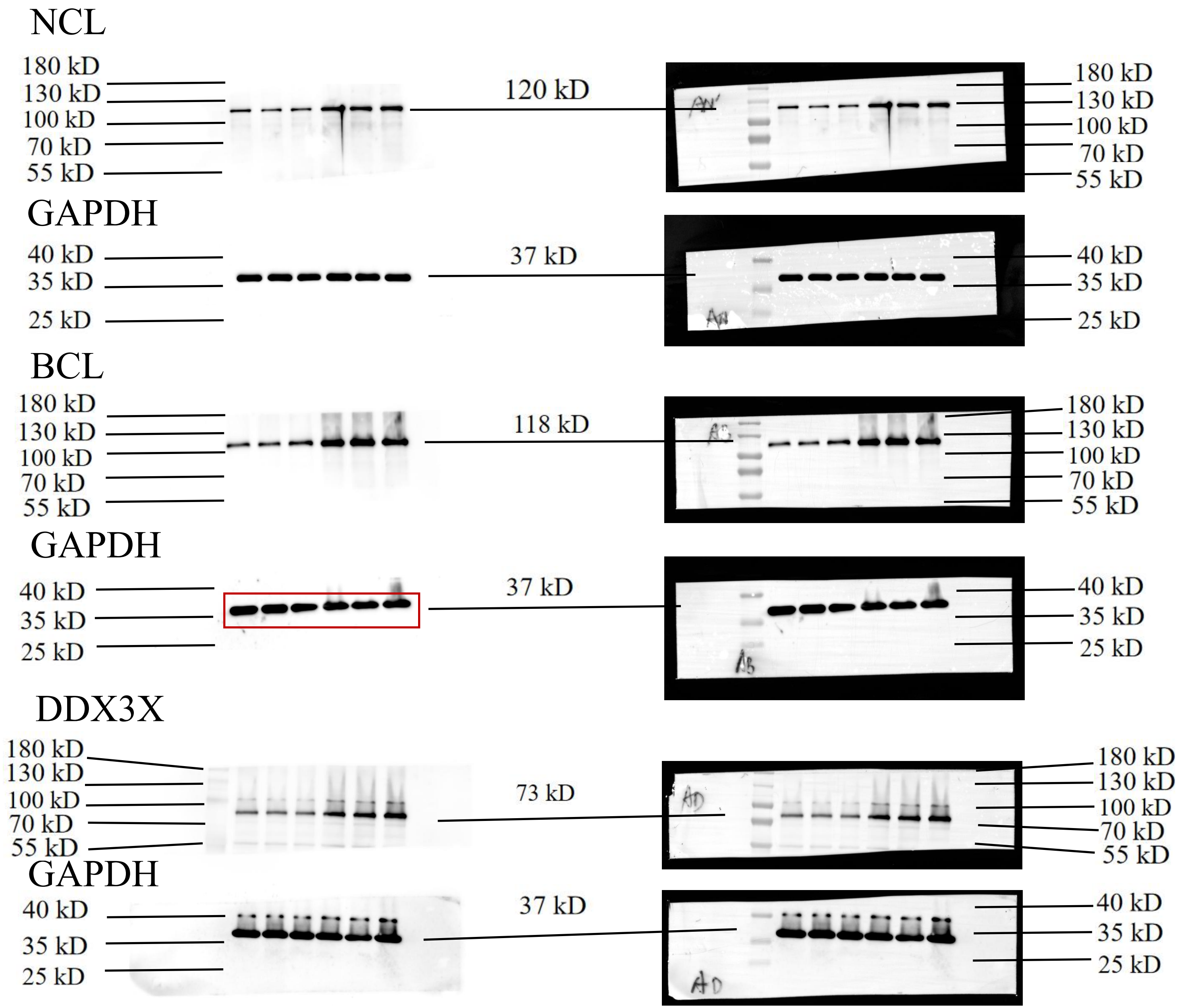
NCL
180 kD
130 kD
100 kD
70 kD
55 kD
120 kD
GAPDH
40 kD
35 kD
25 kD
37 kD
BCL
180 kD
130 kD
100 kD
70 kD
55 kD
118 kD
GAPDH
40 kD
35 kD
25 kD
37 kD
DDX3X
180 kD
130 kD
100 kD
70 kD
55 kD
73 kD
GAPDH
40 kD
35 kD
25 kD
37 kD

Figure 5C
n=3

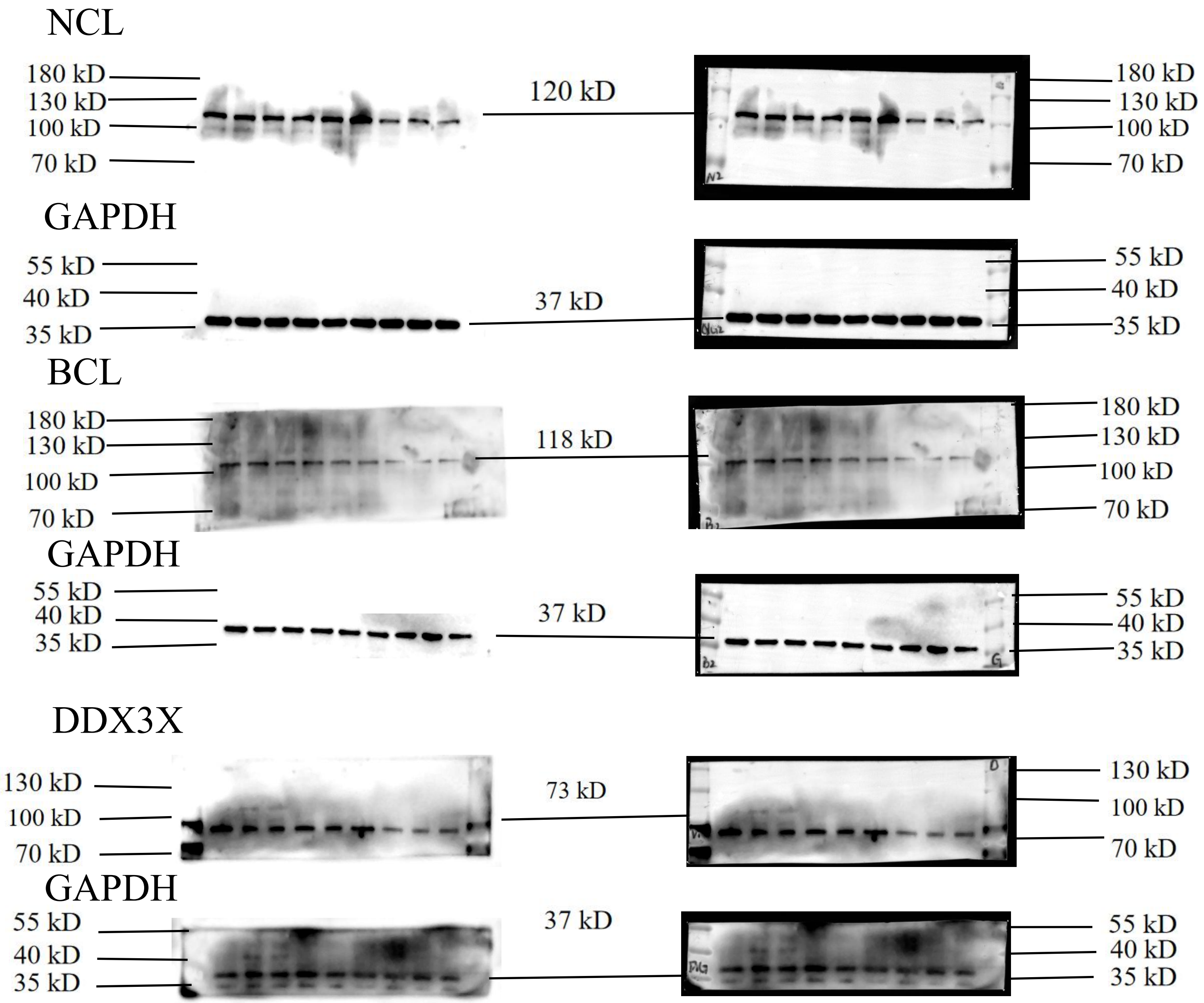

NCL
180 kD
130 kD
100 kD
70 kD
120 kD
GAPDH
55 kD
40 kD
35 kD
37 kD
BCL
180 kD
130 kD
100 kD
70 kD
118 kD
GAPDH
55 kD
40 kD
35 kD
37 kD
DDX3X
130 kD
100 kD
70 kD
73 kD
GAPDH
55 kD
40 kD
35 kD
37 kD

Figure 5E (siDDX3X)

n=3

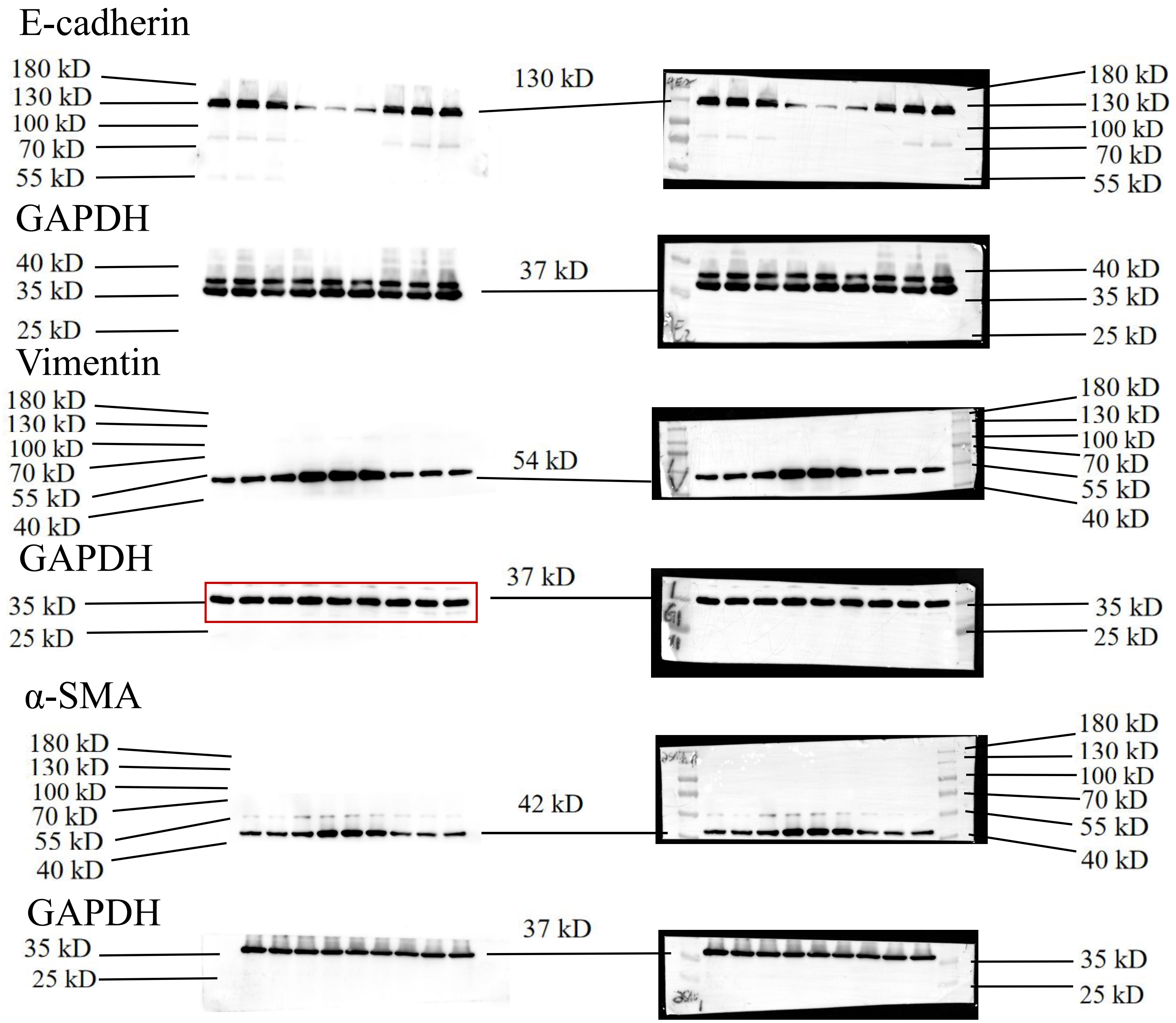

Figure 5E (siBCLAF)

n=3

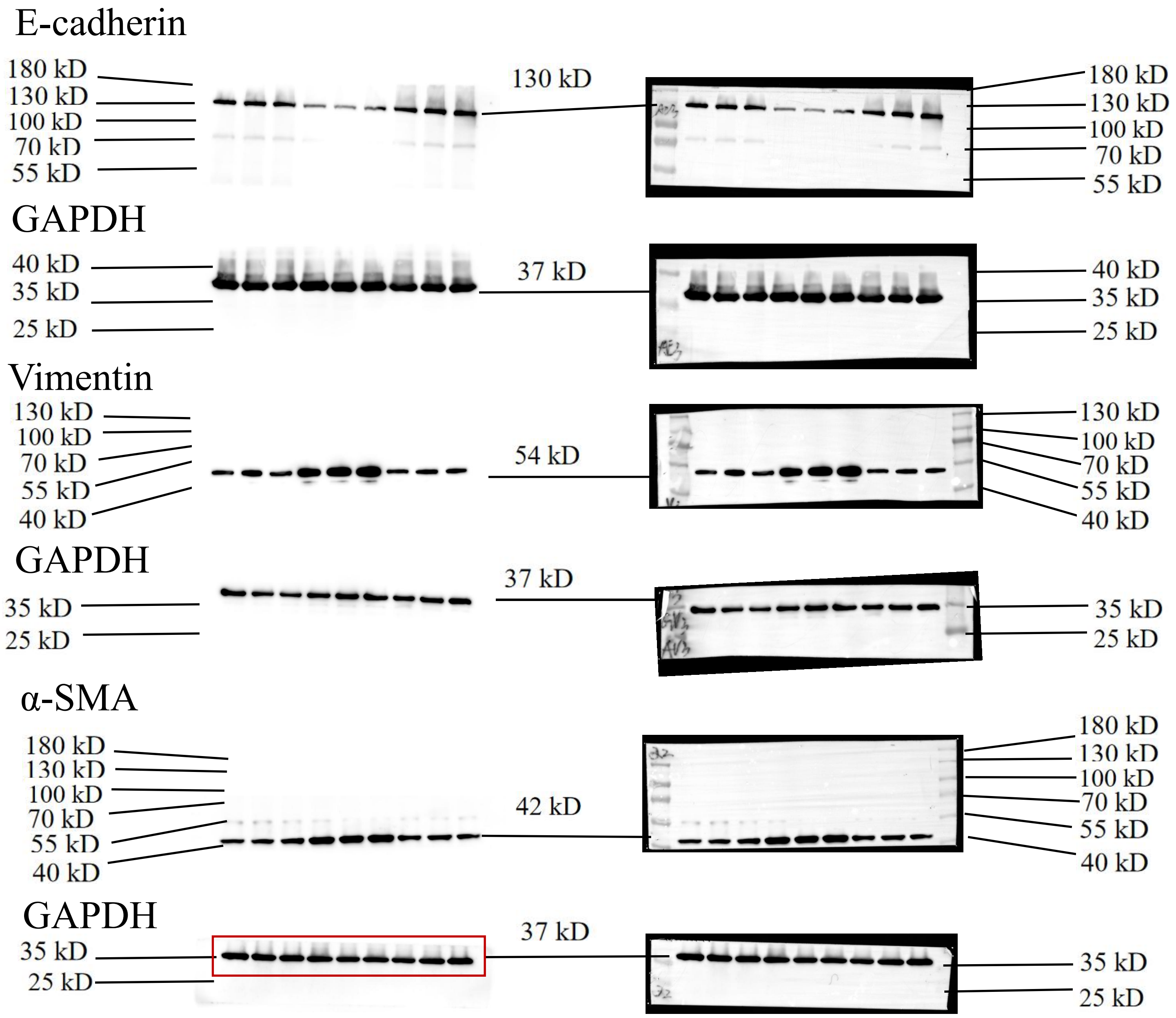

Figure 5E (siNCL)

n=3

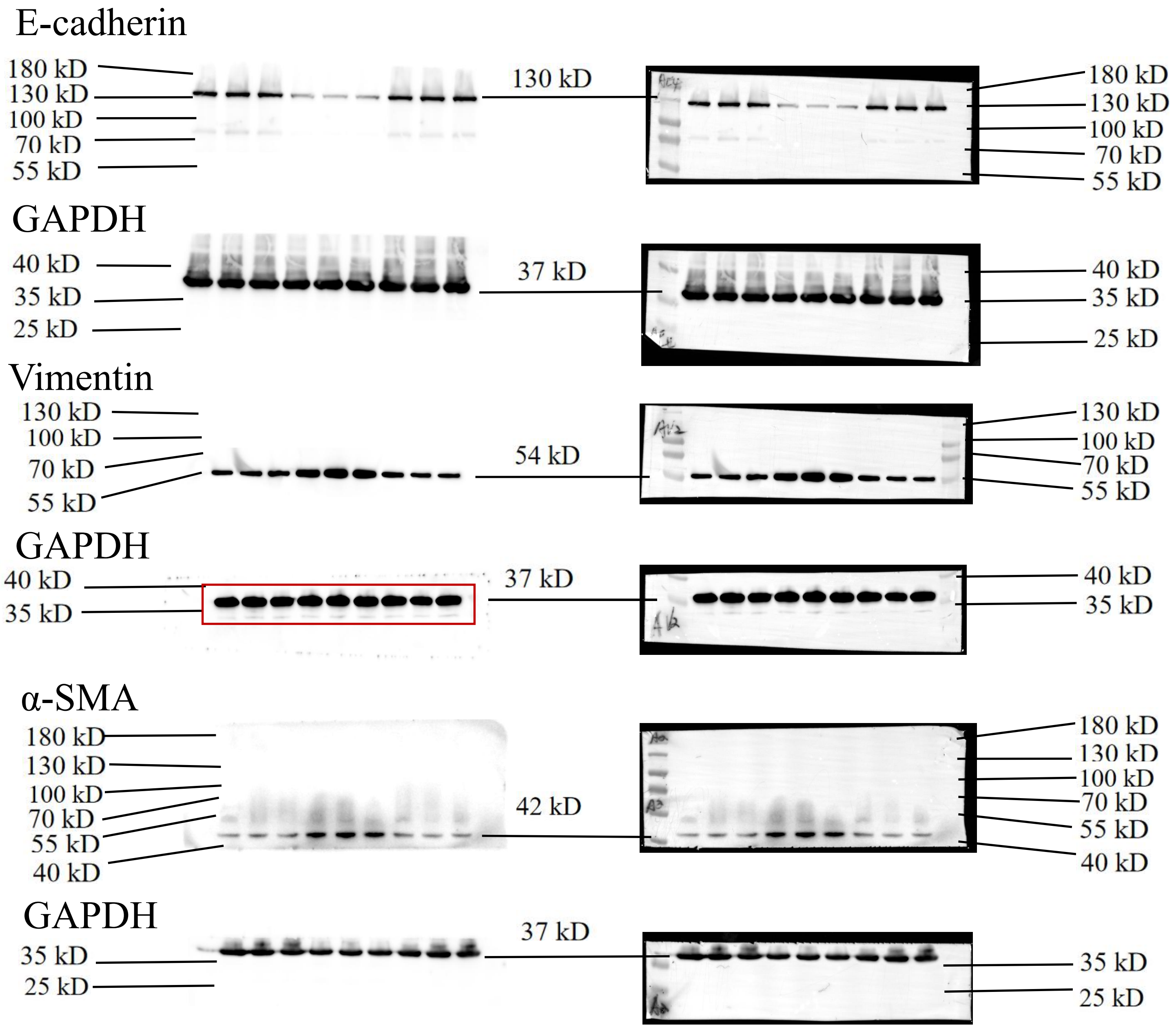

Figure 6A
n=3
Pan-lactylation

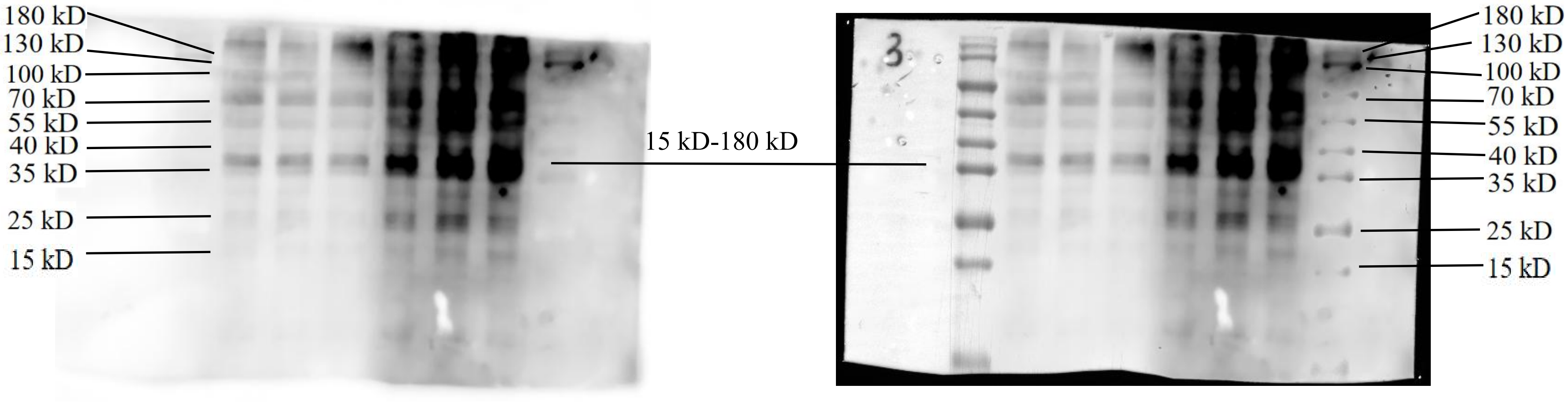

GAPDH

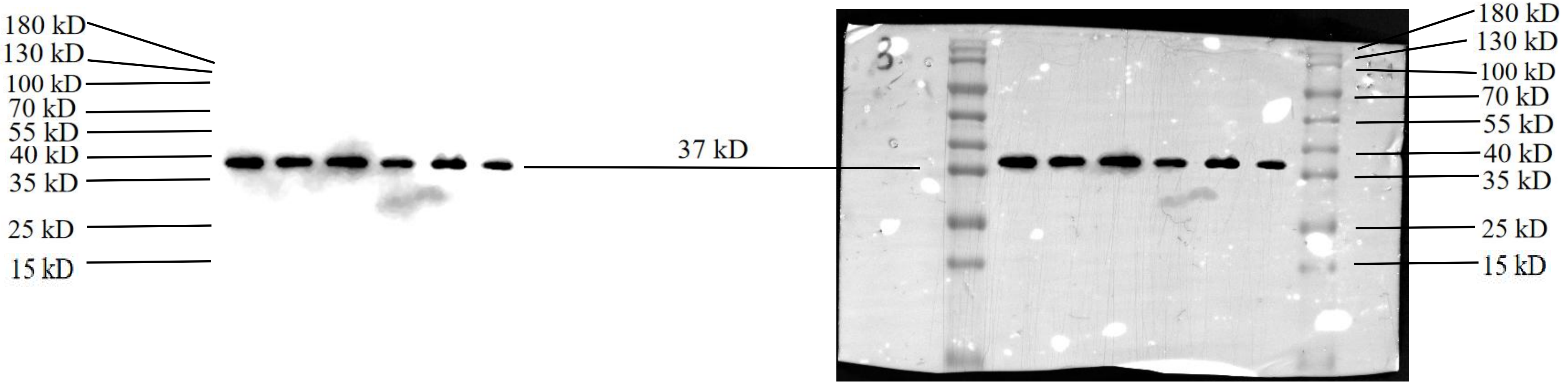

Figure 6B

n=3

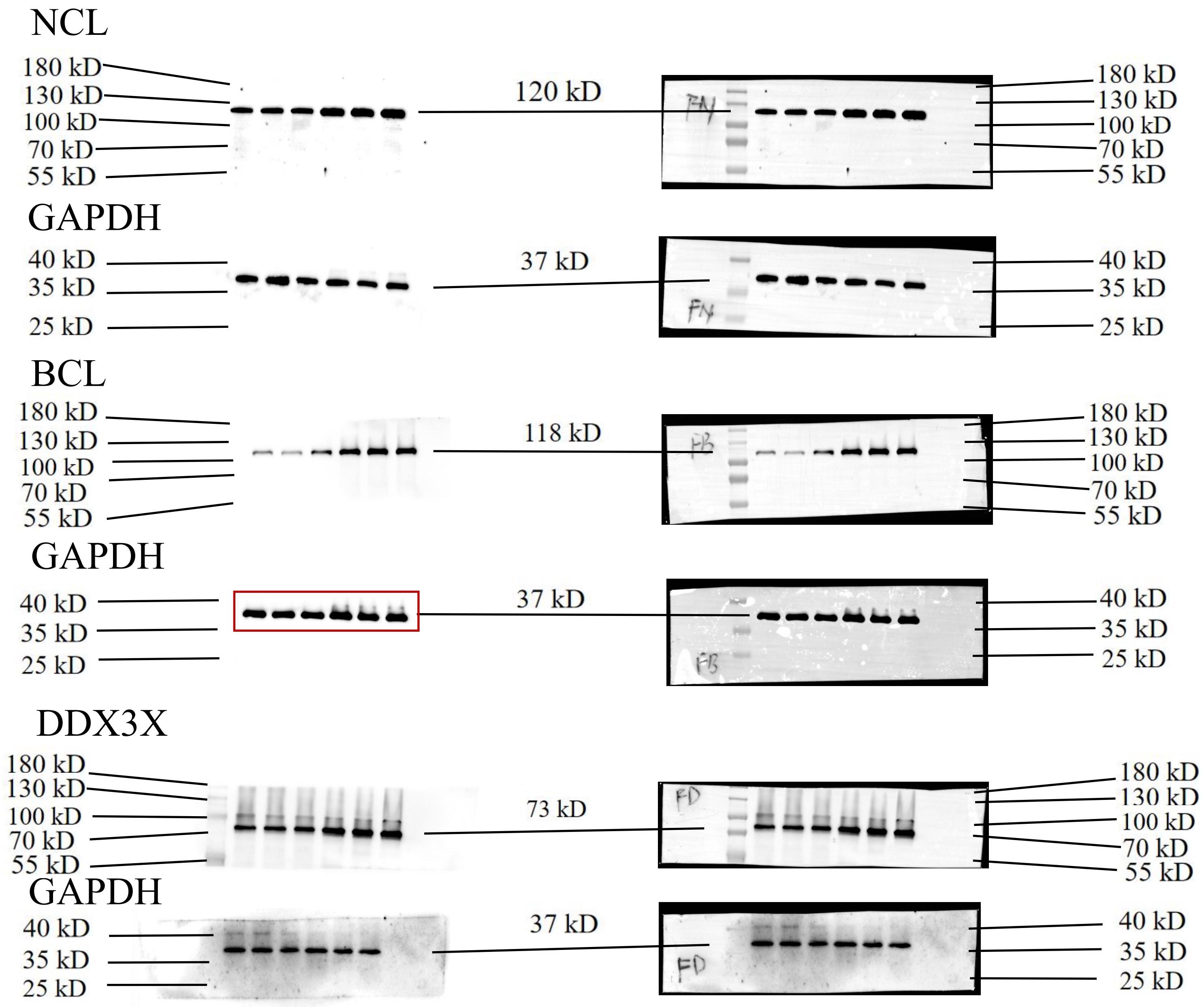
NCL
180 kD
130 kD
100 kD
70 kD
55 kD
120 kD
FN
GAPDH
40 kD
35 kD
25 kD
37 kD
FN
BCL
180 kD
130 kD
100 kD
70 kD
55 kD
118 kD
FB
GAPDH
40 kD
35 kD
25 kD
37 kD
FB
DDX3X
180 kD
130 kD
100 kD
70 kD
55 kD
73 kD
FD
GAPDH
40 kD
35 kD
25 kD
37 kD
FD

Figure 6C

n=3

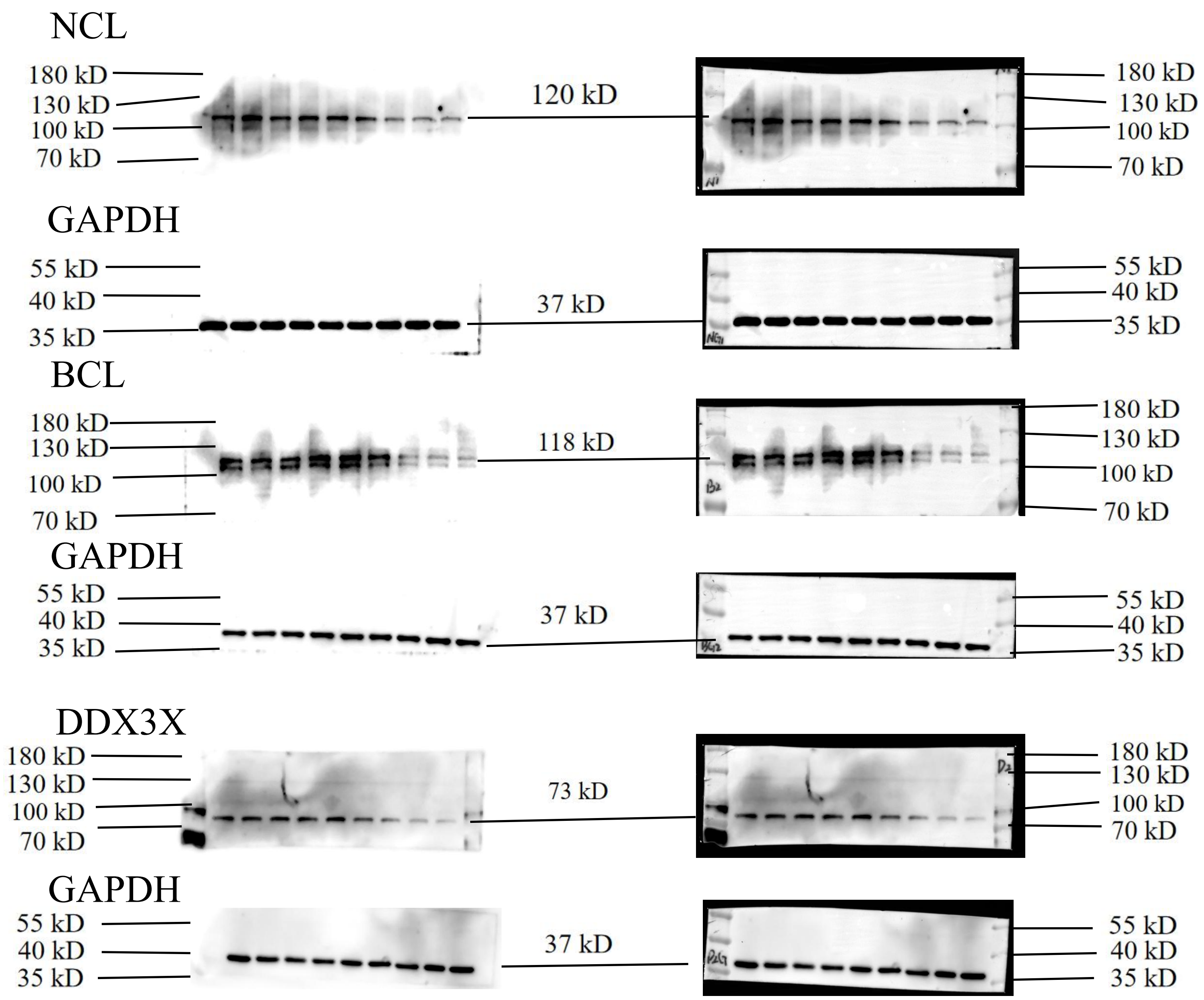
NCL
180 kD
130 kD
100 kD
70 kD
120 kD
180 kD
130 kD
100 kD
70 kD
GAPDH
55 kD
40 kD
35 kD
37 kD
55 kD
40 kD
35 kD
BCL
180 kD
130 kD
100 kD
70 kD
118 kD
180 kD
130 kD
100 kD
70 kD
GAPDH
55 kD
40 kD
35 kD
37 kD
55 kD
40 kD
35 kD
DDX3X
180 kD
130 kD
100 kD
70 kD
73 kD
180 kD
130 kD
100 kD
70 kD
GAPDH
55 kD
40 kD
35 kD
37 kD
55 kD
40 kD
35 kD

Figure 6E (siDDX3X)

n=3

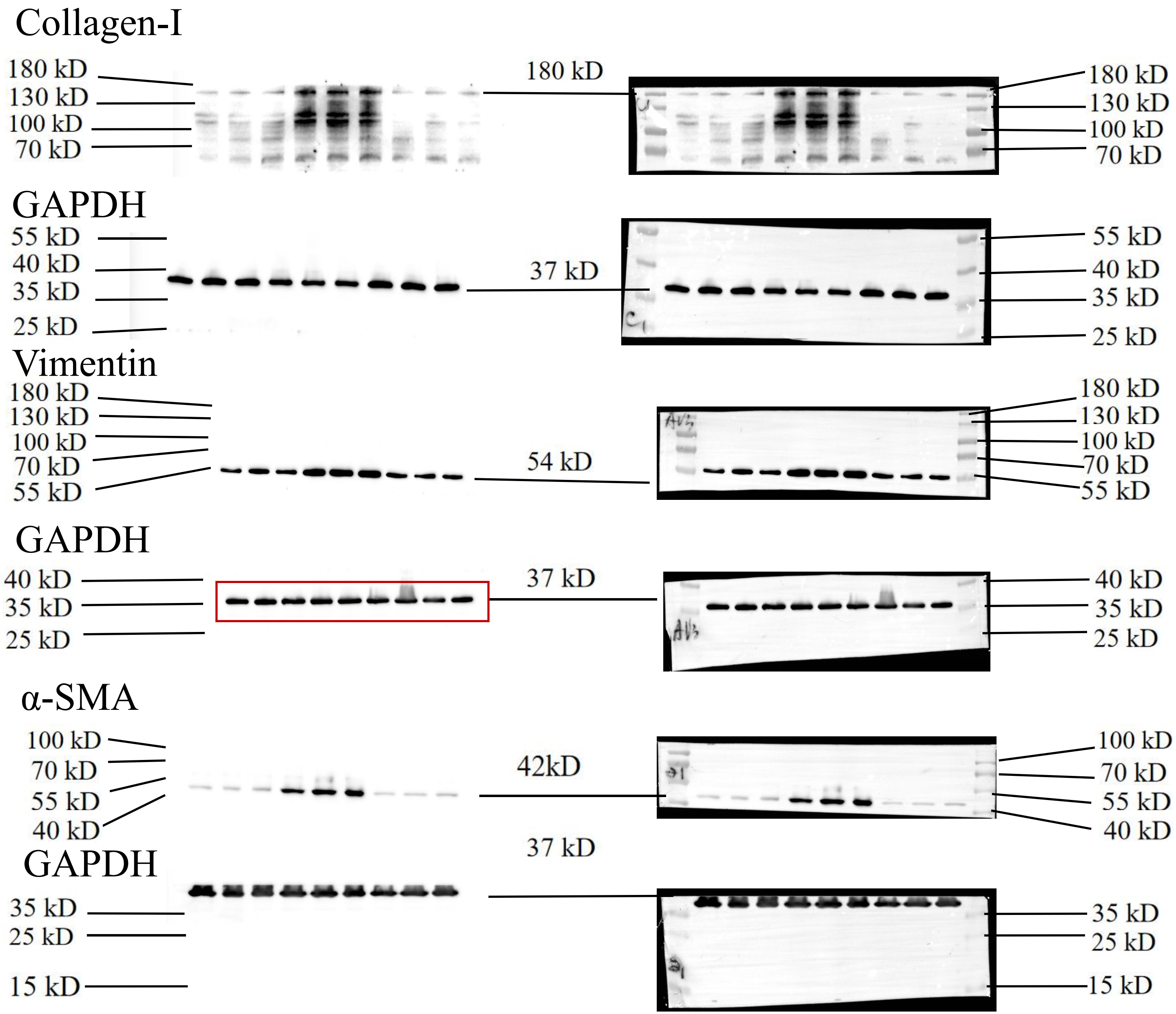

Figure 6E (siBCLAF)

n=3

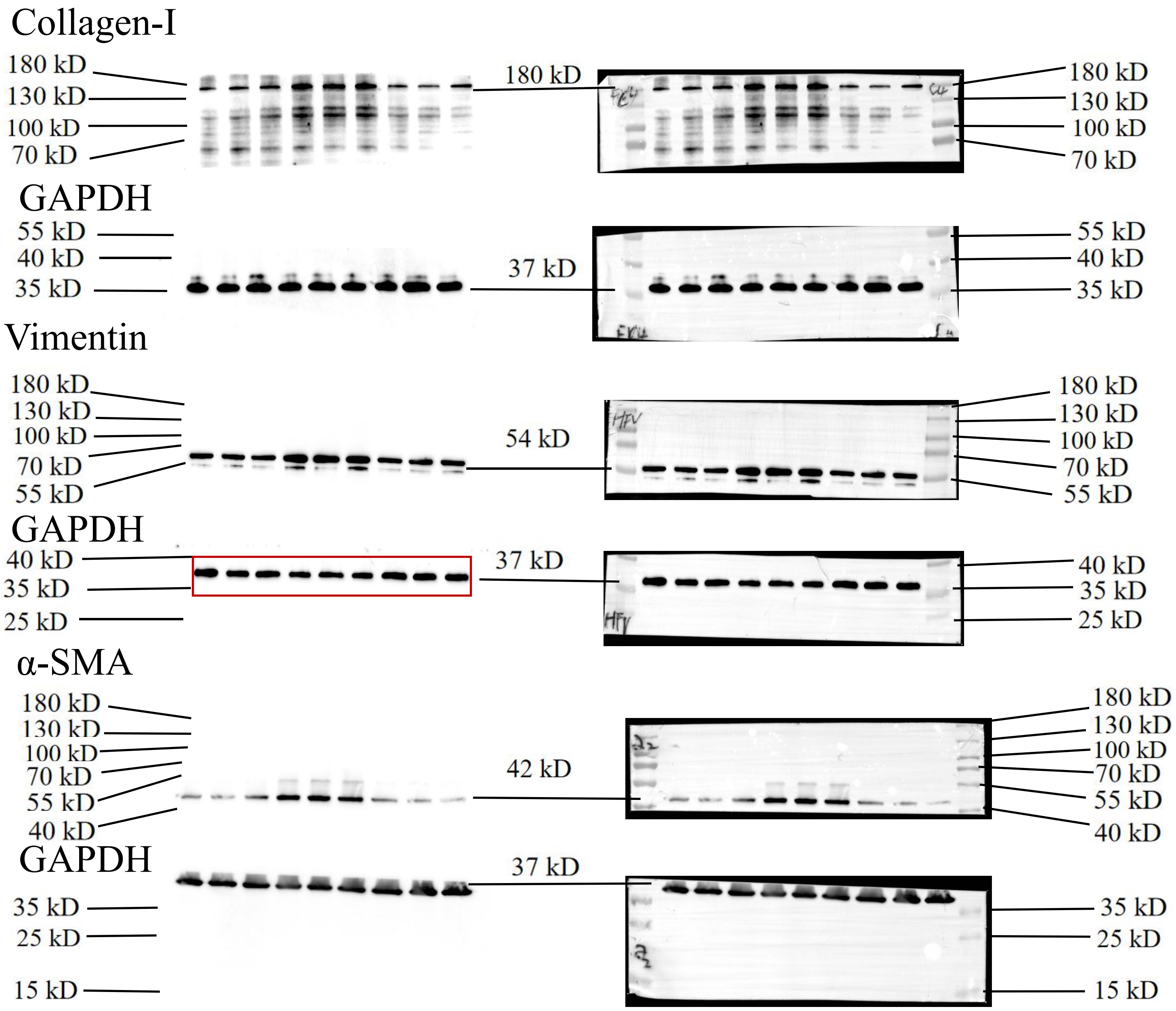

Figure 6E (siNCL)

n=3

Collagen-I

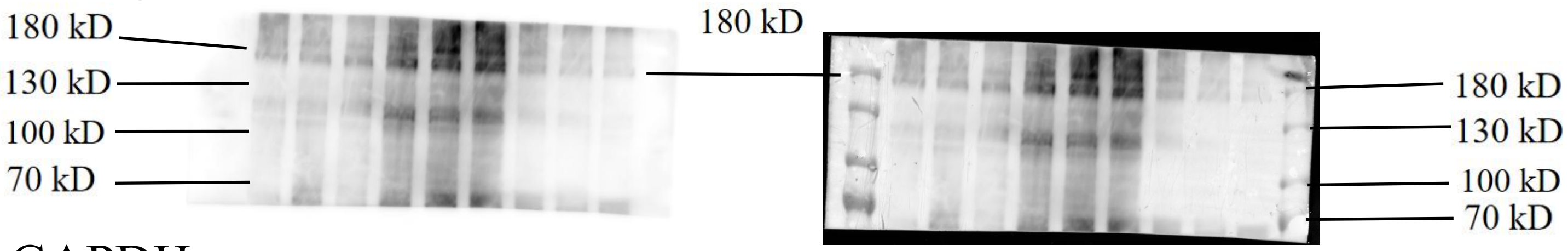

GAPDH

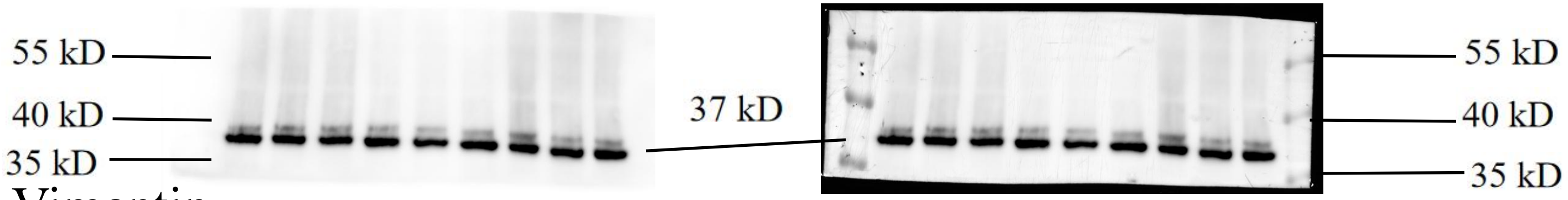

Vimentin

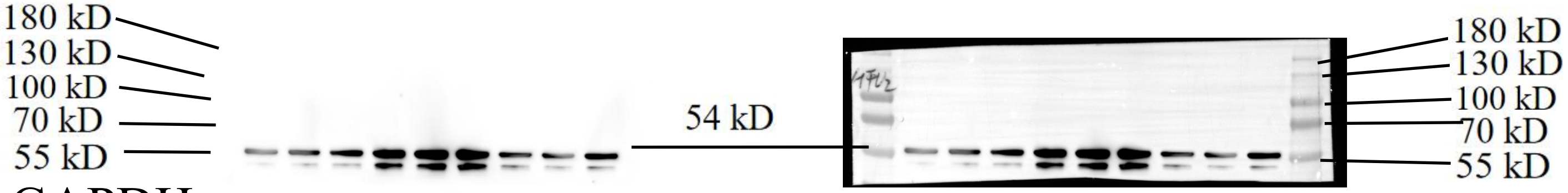

GAPDH

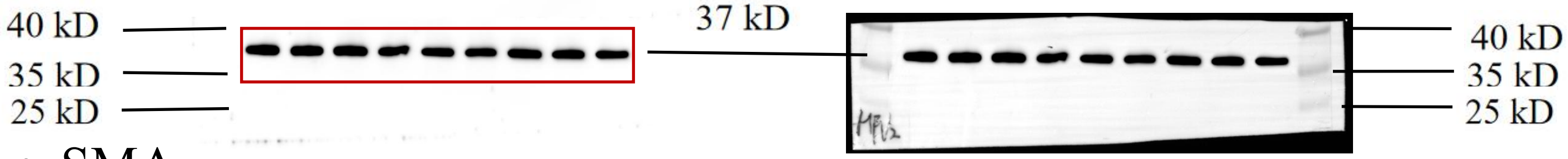

α-SMA

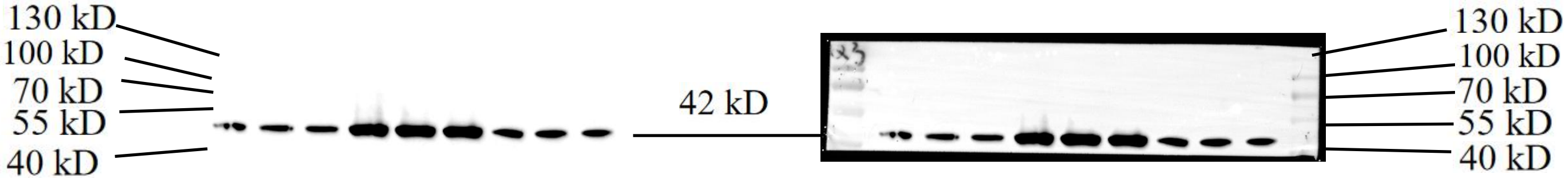

GAPDH

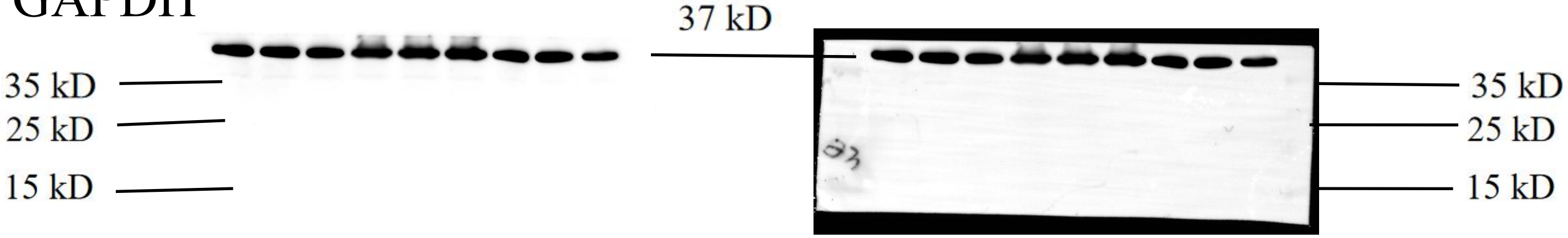

Supplement: Supplementary file 1 [file 12931_2026_3783_MOESM1_ESM.pdf]
